# A neoepitope derived from a novel human germline *APC* gene mutation in familial adenomatous polyposis shows selective immunogenicity

**DOI:** 10.1371/journal.pone.0203845

**Published:** 2018-09-26

**Authors:** Snigdha Majumder, Rakshit Shah, Jisha Elias, Yogesh Mistry, Karunakaran Coral, Priyanka Shah, Anand Kumar Maurya, Bharti Mittal, Jason K. D’Silva, Sakthivel Murugan, Lakshmi Mahadevan, Rekha Sathian, V. L. Ramprasad, Papia Chakraborty, Ravi Gupta, Amitabha Chaudhuri, Arati Khanna-Gupta

**Affiliations:** 1 MedGenome Labs Pvt. Ltd., Bangalore, India; 2 KCHRC, Muni Seva Ashram, Goraj, Gujarat, India; 3 MedGenome Inc., Foster City, CA, United States of America; University of Kentucky, UNITED STATES

## Abstract

Familial adenomatous polyposis (FAP) is an inherited condition arising from genetic defects in the Adenomatous polyposis coli (*APC*) gene. Carriers with mutations in the *APC* gene develop polyps in the colon and rectum which if not managed, transition into colon cancer. In this study, we identified a novel germline mutation in the *APC* gene in members of an FAP-affected (Familial adenomatous polyposis) family. This unique heterozygous variant (c.735_736insT; p.Ser246PhefsTer6) was identified in ten out of twenty six family members, ranging in age from 6 to 60 years. Polyps were detected in six of the ten individuals (35–60 years) carrying this mutation. The remaining four members (6–23 years) remain polyp free. A significant fraction of FAP affected individuals eventually develop colon cancer and therapeutic interventions to prevent cancer progression remain elusive. To address this issue, we sought to determine if peptides derived from the novel APC mutation could induce a cytotoxic T cell response, thereby qualifying them as vaccine candidates. Peptides harboring the variant amino acids were first interrogated *in silico* for their immunogenicity using a proprietary neoepitope prioritization pipeline, OncoPept*VAC*. A single 9-mer peptide was predicted to be immunogenic. Remarkably, CD8^+^ T cells isolated from either an FAP^+^/ APC^mut^ individual, or from a FAP^-^/ APC^mut^ individual, failed to respond to the peptide, whereas those from either an unaffected family member (FAP^-^/ APC^wt^) or from healthy unrelated donors, showed a robust response, suggesting that CD8^+^ T cells from individuals carrying this germline APC mutation have been tolerized to the mutation. Furthermore, experimental testing of six additional reported *APC* gene mutation-derived peptides revealed one of the six to be immunogenic. While not all APC mutant peptides are inmmunogenic, a few qualify as vaccine candidates offering novel treatment opportunities to patients with somatic *APC* gene mutations to delay/treat colorectal cancer.

## Introduction

Familial Adenomatous Polyposis (FAP, OMIM#175100), a type of familial colorectal cancer (CRC), is characterized by the manifestation of adenomatous polyps (typically 100–1000) in the colon and rectum at an early age (mean age of 16), which if left untreated, leads to aggressive and fatal tumors by the age of 40 years[1][2][3]. FAP is caused by autosomal dominant inheritance of germ line mutations in the Adenomatous polyposis coli (*APC*) gene, a well characterized tumor suppressor gene[4]. The *APC* gene is composed of 15 exons, is located on chromosome 5q21-q22 and encodes a protein of 2843 amino acid residues harboring multiple domains[4]. In mammals, APC is expressed in most fetal tissues and in adult epithelial cells[5]. Functionally, the APC protein is an antagonist of the Wnt/ β-catenin signaling pathway, which regulates stem cell pluripotency and cell fate decisions during development. By forming the so called “destruction complex” in combination with glycogen synthesis kinase ß3 (GSK ß3) and Axin, APC induces rapid degradation of β-catenin preventing its translocation into the nucleus. Loss of function of APC leads to the translocation of β-catenin into the nucleus and activation of a transcriptional program, via the TCF/LEF transcription factor, leading to the expression of downstream targets, which include oncogenes such as c-Myc, and other cellular programs regulating cell proliferation and survival[6][7][8].

*APC* is the most commonly mutated gene in colorectal cancer. About 60% of adenomas and carcinomas harbor mutations in the *APC* gene [9]. Over 1500 mutations have been identified in families with both the classic and attenuated types of FAP. More than 60% of these mutations have been mapped to a region in the protein referred to as the Mutation Cluster Region (MCR) located in exon 15 between codons 1284 and 1580 involved in β-catenin downregulation. This region is often lost in the mutated APC protein resulting in the loss of its tumor suppressor function [10][11].

There are currently no curative treatments for FAP and surgical removal of polyps remains the mainstay. However, polyps often return, sometimes in larger numbers and can transform to CRC, if left untreated [12]. The overall 5 years survival rate for FAP-transformed colorectal cancer has been estimated to be 54.4% [13]. Once FAP transforms to CRC, antibodies blocking EGFR and VEGF signaling can prolong survival in a subset of CRC patients [14].

In the last five years, therapies aimed at restoring or enhancing the host’s immune response to treat cancers have gained momentum [15]. Cancer immunotherapy triggers a patient’s immune system to destroy tumor cells by recognizing tumor-derived neoantigens [16][17]. Cancer immunotherapy drugs, such as checkpoint inhibitors, have improved the overall survival of patients with advanced stage cancers, particularly melanoma, non-small cell lung cancer, head and neck cancer and renal cancer [18][19][20]. Cytotoxic T cells recognize tumors as foreign because the latter express tumor-specific neoantigens derived from genetically altered proteins expressed by the tumor cells. These neoantigens are presented on the tumor cell surface as peptides bound to class I and II major histocompatibility complexes (MHC). This MHC-I-peptide complex is recognized by CD8^+^ T cells, which in turn induce tumor cell death by apoptosis [21][22]. In the past few years, attempts have been made to harness the ability of neoantigens to elicit a cytotoxic T cell response to eliminate tumors [23]. The idea of using cancer vaccines as monotherapy or in combination with other cancer immunotherapy drugs has been given impetus by the positive outcomes of two recent clinical trials [24].

In the present study, we used immune cells from both unaffected and affected individuals to demonstrate immunogenicity of a peptide derived from the mutant *APC* gene from an FAP family in an *in vitro* CD8^+^ T-cell activation assay. We show differential immunogenicity of this peptide in carriers of the mutant *APC* gene compared to normal healthy individuals, suggesting central tolerance to the mutation. This is the first report of an immunogenic peptide derived from a germline mutation in the *APC* gene associated with a pre-cancerous condition (FAP). Furthermore, using the same CD8^+^ T cell activation assay and peptides derived from six frequently occurring *APC* gene mutations, we showed one out of the six peptides to be immunogenic, when tested in normal healthy individuals harboring the appropriate HLAs. In sum, our observations suggest that while not all APC mutant peptides are immunogenic, a library of empirically confirmed ones could qualify as vaccine candidates designed to target second hit somatic mutations in the *APC* gene that drive the formation of colon polyps. This approach promises novel treatment opportunities for FAP patients with somatic *APC* gene mutations to delay and/or treat colorectal cancer.

## Methods

### Ethical approval

All procedures performed in studies involving human participants were in accordance with the ethical standards of the institution (KCHRC, Goraj, India) and with the 1964 Helsinki declaration and its later amendments.

### Study subjects

**Fig 1:**
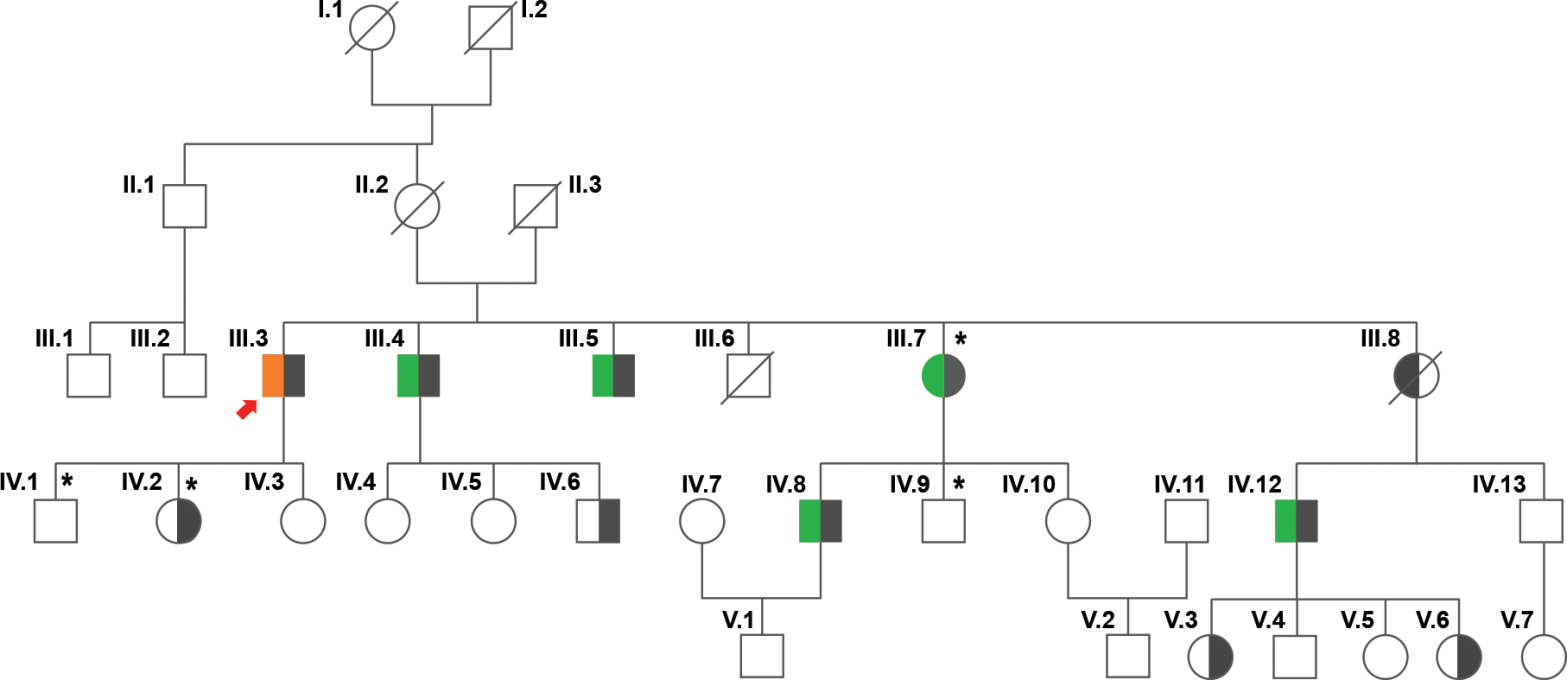
Pedigree of the FAP affected family. The proband (indicated by the red arrow) was diagnosed with FAP and surgically treated for polyp removal and was demonstrated to have a novel *APC* gene mutation. Five additional members of the proband’s extended family were diagnosed with FAP. Biopsy results showed that polyps detected in all affected family members were nonmalignant. Four family members had no polyps but had the *APC* gene mutation. The remaining family members were normal. Color Key: black box: *APC* gene mutation; green box: diagnosed with polyps; and orange box: diagnosed with oral cancer and polyps. A slash through the shape indicates a deceased member. Roman numerals indicate generations. * indicates donors whose PBMC samples have been used for the *in vitro* T cell activation assay. Note: Sample for individual V.4 could not be collected.

The proband (Fig 1, III.3, black arrow) was diagnosed with FAP at Kailash Cancer Hospital and Research Centre (KCHRC) Goraj, India, and was surgically operated for the removal of multiple polyps following colonoscopy. Five other affected members of his extended family were also diagnosed with FAP. Following Ethics committee approval at KCHRC, blood samples were collected by venipuncture in EDTA tubes from all 26 members of this family (see pedigree, Fig 1). All study subjects signed an informed consent prior to sample collection.

### Mutational analysis

Genomic DNA was extracted from the blood samples using a Qiagen kit (QIAsymphony DNA midi Kit, Cat # 931255) using recommended protocols (Qiagen, Germantown, MD, USA). To identify the causal gene variant in this family, targeted next generation sequencing (NGS) was performed on 3 individuals (Fig 1, III.3, III.4, and III.7). Libraries were prepared with the extracted DNA using a Kapa Biosystems kit as per manufacturers instruction (Massachusetts, USA), and hybridized on a Roche Nimblegen custom designed 7MB panel (Details of this panel are given in the S1 Table) following the manufacturer’s protocol. Libraries were then subjected to paired-end sequencing on an Illumina HiSeq 2500. NGS data for the three FAP affected family members has now been submitted to EBI ENA database (https://www.ebi.ac.uk/ena/submit/sra/#home) with Accession ID: PRJEB27848. All 26 family members were screened for the mutation in the *APC* gene by Sanger sequencing using standard protocols on an ABI 3730xl instrument using the following oligos:

APC.e09.5i: 5’ CGTACTGGAGGTTATGAAGTG 3’;

APC.e09.3i: 5’ AGAGAAATGACAGCACATTG 3’

### Bioinformatics analysis

The raw NGS data obtained was subjected to adapter trimming using the Fastq-Mcf tool followed by alignment to the human reference genome (hg19) using the BWA aligner. The best practices GATK germline variant calling workflow was used for calling SNPs and short INDELS. The aligned reads were sorted and de-duplicated using Picard and then realignment and recalibration was performed using GATK. Large insertions and deletions were called using the Pindel program. Bedtools program was used to calculate target region coverage. The variants obtained were annotated using MedGenome’s in-house variant annotation toolkit (VariMAT). The VariMAT pipeline performs deep annotation which includes gene, disease and common polymorphism annotation. Disease annotation was performed against HGMD, OncoMD (a proprietary in-house data base), OMIM, GWAS and Clinvar while common polymorphism annotation was obtained against the 1000-Genome, ExAC, dbSNP, ESP, 1000Japanese, dbSNP databases.

### HLA typing

HLA typing of 1 unaffected (Fig 1, III.7) and 2 affected (APC mutation positive) family members (Fig 1, IV.2 and IV.9) was performed. Amplification of the HLA locus-specific regions was performed by long range PCR using genomic DNA isolated from blood. The assay uses proprietary HLA locus-specific primers supplied in the GenDx amplification kit (Utrecht, The Netherlands). Single indexed libraries were prepared from the HLA amplicons using the KAPA Biosystems kit (Massachusetts, USA). The libraries were then sequenced on an Illumina HiSeq4000 HT system. Data was analyzed by the GenDx software.

### Neoepitope prediction

Neoepitope prediction and prioritization was performed using the proprietary OncoPept*VAC* neoepitope prediction pipeline. Briefly, the prediction pipeline combines a novel TCR-binding prediction algorithm (Manuscript submitted) with other commercial tools for peptide-MHC binding (NetMHCcons 1.1, http://www.cbs.dtu.dk/services/NetMHCcons/ [25], the peptide-processing module of IEDB (netChop 3.1 http://www.cbs.dtu.dk/services/NetChop/) and peptide TAP binding (http://tools.iedb.org/processing/) to select peptides that are predicted to be presented by antigen presenting cells and are likely to bind to the T cell receptor (TCR).

### T cell activation assay

The naïve CD8^+^ T cell activation assay was performed as described previously [26]. Briefly, peripheral blood mononuclear cells (PBMCs) were isolated from heparinized blood collected from consented donors (Fig 1, III.7, IV.2, IV.9) using a standard Ficoll gradient (GE Healthcare, USA). The isolated PBMCs were then frozen and stored in liquid nitrogen for later use. Peptides derived from the identified *APC* gene mutation (S3 Table), were synthesized at JPT Peptide Technologies, (Berlin, Germany). All cytokines used in our assay were procured from Peprotech, Rehovot, Israel. Monocytes from thawed PBMCs were isolated by the adherence method, differentiated into dendritic cells and pulsed with wild type or mutant peptides. Naïve CD8^+^ T cells were isolated using the MACS cell separation method, (Miltenyi Biotec, Cologne, Germany) and co-cultured with peptide- pulsed dendritic cells. After culturing for 10 days, T cells were restimulated with peptide loaded PBMCs for 48 hrs and then intracellular cytokine staining was performed after treating the cells for 5 hours with using Brefeldin A (BD, Cat No. 51-2301KZ), fixed and permeabilized using BD Lysis solution (Cat No. 349202) and Perm2 (BD, Cat No. 347692) and stained with CD3 (Biolegend Cat No. 300408), CD8 (BD Cat No. 34105) IFNγ (Biolegend Cat No. 502512) and TNFα (Biolegend, Cat #: 50290), and the BD Fast immune CD8 intracellular cytokine kit (Cat. No. 346048). Stained cells were analyzed in a Beckman Coulter Navios Flow Cytometer (Beckman Coulter, USA) to detect the expression of the T cell activation markers, IFNγ and TNFα.

A second CD8+ T cells activation assay was performed as follows: PBMCs were treated with 5μM of synthetically synthesized peptides (JPT, Germany) in the presence of IL-15 and IL-2. Every 3 days, the media was replaced with fresh media containing 10IU of IL-2 and 10ng/ml IL-15. On the 7^th^, 14^th^ and 21^st^ days of incubation, fresh peptides were added to the respective wells. On day 22, cells were treated with Brefeldin A for 5 hours, cells were fixed, permeabilized using BD Lysis solution and Perm2 and stained with antibodies. Stained cells were analyzed in a Beckman Coulter Navios Flow Cytometer (Beckman Coulter, USA) to detect the expression of the T cell activation markers, IFNγ and TNFα. Data was analyzed using Kaluza software (Beckman Coulter). All experiments were performed twice in triplicate. Statistical analysis using student’s t-test was performed, where appropriate, and a *p* value of ≤0.05 was considered to be significant.

## Results

### Identification of a novel germline mutation in the *APC* gene in an FAP-affected family

The proband (Fig 1, III.3) presented with weight loss and changes in bowel movement in 2014 and was diagnosed with FAP based on clinical symptoms and presentation. Colonoscopy confirmed the presence of multiple polyps in the colon of this patient. The polyps were subsequently removed surgically. This patient was previously diagnosed with oral cancer and underwent treatment with chemotherapy and radiation therapy in 2013. On further clinical investigation, five other members of the proband’s extended family were diagnosed with FAP. With the exception of one individual, affected family members were operated to remove multiple polyps in the colon and rectum (Table 1). Biopsy results showed that the polyps were nonmalignant in all affected members. A pedigree chart of this family is shown in Fig 1.

**Table 1:** Clinical data of FAP family members. The sample ID of each individual follows the pedigree illustrated in Fig 1. PBMCs were used from the donors highlighted in green for an *in vitro* CD8^+^T cell activation assay to test the immunogenicity of the mutant APC peptide.

Sample ID	Age	APC Mutation	Carcinogenic exposure	Diagnosis	Surgery
II.1	41	Absent	None		
III.1	9	Absent	None		
III.2	16	Absent	None		
III.3	52	Present (Het)	tobacco chewing	FAP, Oral Cancer	Yes
III.4	54	Present (Het)	Pan masala with tobacco	FAP	Yes
III.5	44	Present (Het)	Pan masala with tobacco	FAP	
III.7	60	Present (Het)	None	FAP	Yes
IV.1	21	Absent	Pan masala with tobacco		
IV.2	23	Present (Het)	None		
IV.3	17	Absent	None		
IV.4	23	Absent	None		
IV.5	18	Absent	None		
IV.6	18	Present (Het)	None		
IV.7	28	Absent	None		
IV.8	35	Present (Het)	Smoking cigarette	FAP	Yes
IV.9	27	Absent	None		
IV.10	31	Absent	None		
IV.11	34	Absent	None		
IV.12	38	Present (Het)	Pan masala with tobacco/ chewing tobacco	FAP	Yes
IV.13	36	Absent	Smoking cigarette		
V.1	4	Absent	None		
V.2	4	Absent	None		
V.3	8	Present (Het)	None		
V.5	12	Absent	None		
V.6	6	Present (Het)	None		
V.7	9	Absent	None		

**Fig 2:**
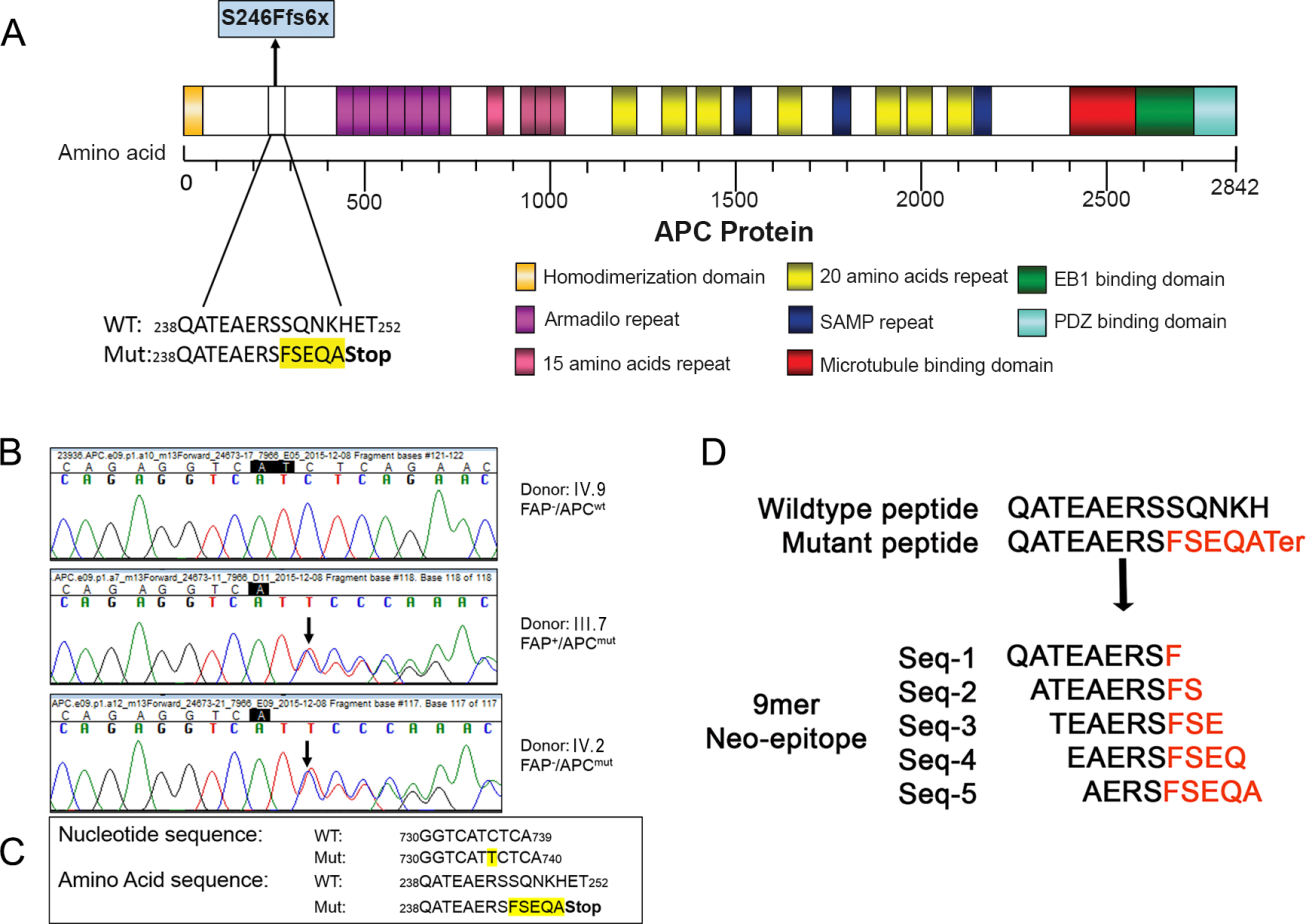
**A. Structure of the 2842 amino acid APC protein (drawn to scale).** The frame-shift mutation (S246Ffs6X) is indicated and the resultant mutant amino acids highlighted in yellow. A number of color coded functional motifs have been illustrated, including the mutation cluster region (MCR) harboring the β-catenin binding domains which are critical for the tumor suppressor function of the APC protein**. B.** Sanger sequencing chromatograms revealing the wildtype and mutant *APC* gene sequences. The mutation involving the insertion of a T between nucleotide 735 and 736 was found in two individuals tested (indicated by a black arrow), IV.8 and IV.6 (middle and bottom panels), but absent in the unaffected individual (VI.4, top panel). **C.** The nucleotide and protein sequences of both wildtype and mutant APC genes are indicated and the mutant sequences highlighted in yellow**. D.** *In silico* prediction of 9 mer-peptides generated from the wildtype and mutant protein sequences. Residues indicated in red are amino acids that are unique to the mutant APC protein.

We analyzed the DNA of the proband (Fig 1, III.3) and two other affected (Fig 1, III.4, III.7) family members on a gene panel consisting of 1961 genes associated with hereditary diseases, including cancer (S1 Table). Samples were sequenced on an Illumina HiSeq2500. Total data generated for each sample exceeded 1.5 GB and more than 90% of the data was above Q30. More than 99% of the reads aligned to the reference genome and all the genes were covered at >99% with an average sequencing depth of 138-175X (S2 Table). Data analysis identified a unique heterozygous variant (chr5:112136981-112136982insT; c.735_736insT; p.Ser246PhefsTer6) in the *APC* gene (S1 Fig) in all three affected family members (Fig 1, III.3, III.4, III.7). The insertion of a single T-residue between nucleotides 735 and 736 in the coding exon of the *APC* gene resulted in a frameshift mutation leading to a truncated protein (p.Ser246PhefsTer6) (Fig 2A). The *APC* gene is the most commonly mutated gene in FAP, and the loss of function variant identified in this family is likely to be pathogenic.

To confirm whether this mutation was present in other family members, we performed Sanger sequencing analysis on all 26 family members. The unique heterozygous *APC* gene mutation was confirmed to be present in 10 out of the 26 family members (Fig 1 and Table 1). All six individuals diagnosed with FAP were confirmed by Sanger sequencing to have this germline mutation. Affected individuals were 35 years or older (35–60 years) when diagnosed with polyps. Genetic counseling and regular colonoscopies have been prescribed for the high- risk individuals who carry this heterozygous *APC* gene mutation, including the four individuals aged 6–23 years, who currently remain polyp free.

### Neoepitope prediction and prioritization

It is well established that the host immune system can eliminate transformed cells early during tumor development by immune surveillance mechanisms [27][28]. The recognition of the tumor as non-self is mediated by tumor-derived neoepitopes, which are immunogenic peptides arising from intracellular proteolytic processing of somatic mutations in protein coding genes. These peptides bind HLA Class I (MHC) proteins and are presented on the surface of antigen presenting cells. Productive engagement of the HLA Class I-bound peptide with the T-cell receptor (TCR) activates naïve CD8^+^ T-cells, transforming them into cytotoxic T cells, which mediate lysis of the neoepitope-expressing tumor cells [29].

To address whether peptides derived from the mutant *APC* gene could evoke a T cell response, we selected three members of this family with the following genotypes: FAP^-^/*APC*^wt^ (unaffected) (Fig1, IV.9); FAP^-^/*APC*^mut^ (no polyps with *APC* gene mutation, Fig 1, IV.2); and FAP^+^/*APC* ^*mut*^ (polyps with *APC* gene mutation, Fig 1, III.7). The *APC* gene mutation status was confirmed in the three selected individuals by Sanger sequencing (Fig 2B and 2C). The HLA type of the three individuals was determined (Table 2). We next generated 9-mer peptides *in silico* from the wild-type and the mutated APC proteins (Fig 2C and 2D and S3A and S3B Table) and predicted their immunogenicity using the OncoPept*VAC* algorithm (Table 3 and S3A and S3B Table). Our analyses revealed that of all the wild-type and mutant peptide pairs analyzed, the mutant peptide QATEAERSF (Table 3, Mutant peptide) was predicted to have a strong binding affinity (IC_50_ 151.47nM and 621.64nM) to both HLA B35:01 and HLA C03:03 respectively, as determined by NetMHCcons 1.1 [25]. In addition, the mutant peptide was predicted to be positive for TCR binding. The corresponding wild-type peptide QATEAERSS (Table 3, Wildtype) on the other hand, had a relatively poor HLA-binding affinity (IC_50_ 14485.65 nM and 28178.94 nM) for the same HLAs. The significant differences in binding affinities between the wild-type and mutant peptides coupled with their predicted positive TCR-binding ability, led us to empirically test their immunogenicity in an *in vitro* CD8^+^ T cell activation assay.

**Table 2:** HLA typing of unaffected and affected family members. Pedigree of the family is shown in Fig 1.

**IV.9 FAP**^**-**^**/APC**^**wt**^		
HLA-A	A*11:01:01:01	A*24:17
HLA-B	B*15:01:01:01	B*35:01:01:02
HLA-C	C*03:03:01:01	C*04:01:01:01
**IV.2 FAP**^**-**^**/APC**^**mut**^		
HLA-A	A*01:01:01:01	A*01:01:01:01
HLA-B	B*35:01:01:02	B*57:01:01
HLA-C	C*04:01:01:01	C*06:02:01:01
**III.5 FAP**^**+**^**/APC**^**mut**^		
HLA-A	A*01:01:01:01	A*24:17
HLA-B	B*15:01:01:01	B*57:01:01
HLA-C	C*03:03:01:01	C*06:02:01:01

**Table 3:** Properties of wildtype and mutant peptides relevant to antigen presentation and T cell receptor binding as accessed by Oncopept*VAC* (see methods for details on the algorithm).

HLA type	Wildtype peptide	Affinity (nM)	TAP score	Processing score	TCR binding
HLA*B35:01	QATEAERS**S**	14485.65	-1.01	0.95	Positive
HLA*C03:03	QATEAERS**S**	28178.94	-1.01	0.95	Positive
	**Mutant peptide**				
HLA*B35:01	QATEAERS**F**	151.47	1.07	1.3	Positive
HLA*C03:03	QATEAERS**F**	621.64	1.07	1.3	Positive

### The mutant APC peptide is immunogenic

Only one pair (*APC* wild type: QATEAERSS; mutant: QATEAERSF), out of the five peptide pairs arising from the APC mutation (c.735_736insT; p.Ser246PhefsTer6) was predicted to be immunogenic by OncoPept*VAC* analysis, while the others four (Fig 2D, Seq 2–5) were predicted (S3A and S3B Table) and tested in our CD8-T cell activation assay to be non-immunogenic (data not shown). To examine the immunogenicity of the prioritized peptide pair (Fig 2D, Seq 1), a CD8^+^ T cell activation assay wherein naïve CD8^+^ T cell-dendritic cells were co-cultured in the presence of either the synthetic wild type (QATEAERSS) or mutant APC peptides (QATEAERSF) (Fig 2C and 2D). The peptides were presented on autologous monocyte derived dendritic cells to the naïve CD8+ T cells isolated from PBMCs from each of the three family members, as well as from an unrelated healthy donor (healthy donor 1). Naïve T cell were considered activated if in response to the peptides, intracellular staining for IFNγ was positive, as measured by FACS analysis.

**Fig 3:**
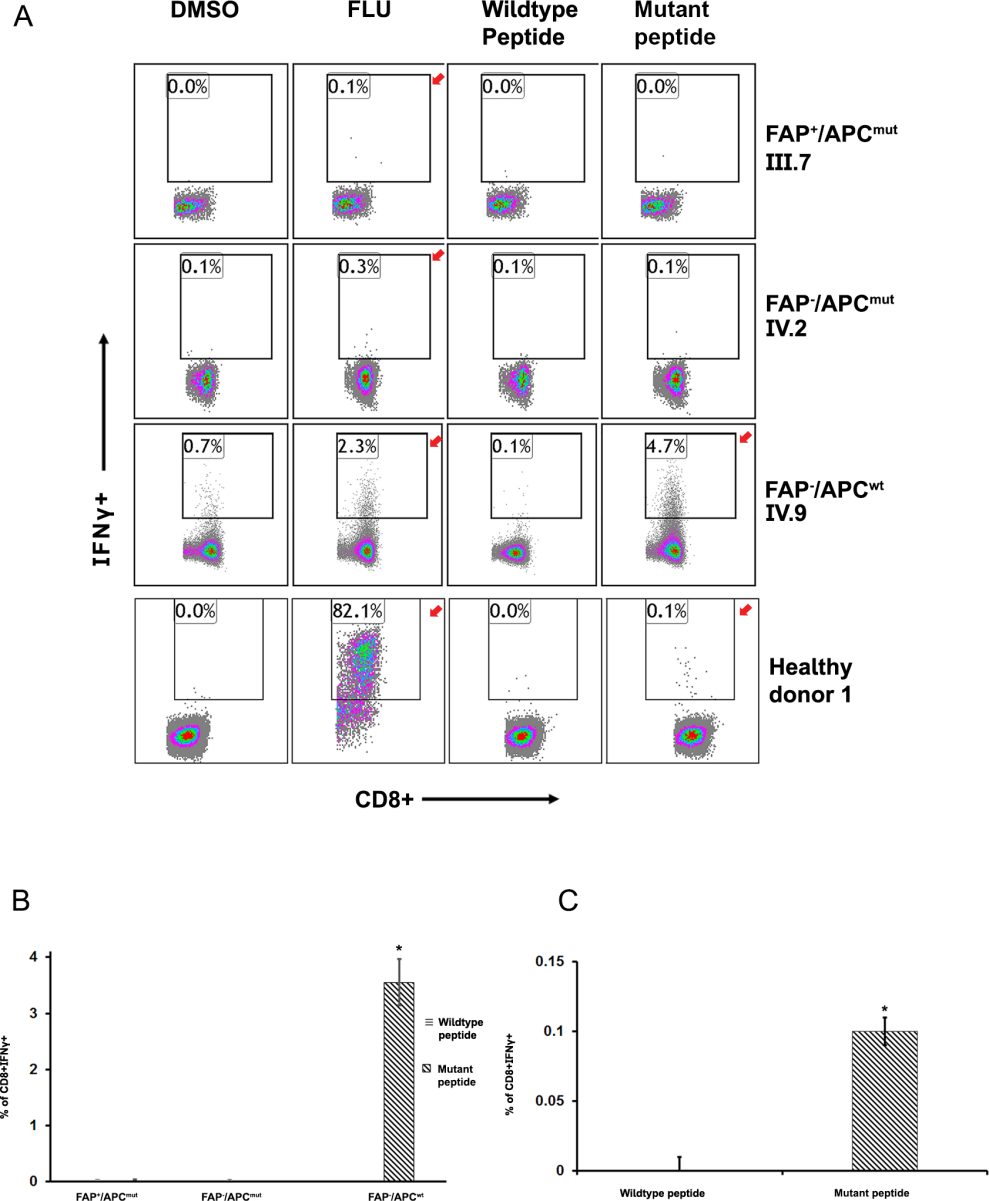
The mutant APC peptide is selectively immunogenic. **A.** Purified CD8^+^ T cells and monocyte-derived DCs from each of the three individuals was tested for antigen-specific T cell activation using wildtype and mutant APC peptides (panels A-C). Donor IV.9 (FAP^-^/APC^wt^) and unrelated healthy donor 1 demonstrated a robust CD8^+^T cell response to the mutant APC peptide compared to the wildtype peptide as measured by IFNγ^+^ positivity (panel 3 and 4 respectively). Donor IV.2 (FAP^-^/APC^mut^, panel 2) and donor III.7 (FAP^+^/APC^mut^, panel 1) showed no increase in IFNγ^+^ cells. Red arrows indicate an upregulation of IFNγ^+^ cells. Positive control CEF pool peptides (FLU) showed upregulation of IFNγ^+^ in all four donors validating cells and reagents used in the assay. **B.** Graphical representation of the Flow cytometry data of FAP family members III.7, IV.2 and IV.9 in Fig 3A (*p = 0.003). **C.** Graphical representation of the Flow cytometry data of unrelated healthy donor 1 in Fig 3A bottom panel. The data plotted are the means of triplicates +/- SD (*p = 0.0009).

A significant increase (3.5-fold change) in the percentage of IFNγ positive cells was observed in an unaffected individual (FAP^-^/APC^wt^, IV.9) in response to the mutant peptide compared to wild-type peptide (Fig 3A and 3B), suggesting that the mutant peptide is capable of eliciting an immune response in an unaffected individual. In contrast, CD8^+^T cells isolated from FAP^+^/ APC^mut^ and FAP^-^/ APC^mut^ individuals failed to respond to either the mutant or to the wild-type APC peptides (Fig 3A and 3B, individuals IV.2, III.7). These observations suggested that the APC-derived mutant peptide was immunogenic only in unaffected individuals lacking the germ line *APC* mutation, while family members carrying this mutation were unresponsive, regardless of polyp development. Furthermore, testing of these peptides in two unrelated healthy donors (healthy donors 1 and 2) led to a strong CD8^+^ T cell response as measured by the upregulation of IFNγ and TNFα positive cells (Fig 3C and S2A, S2B and S3 Figs). Lack of a CD8^+^T cell response in individuals carrying the *APC* germline mutation is likely due to the development of central immunologic tolerance to this mutation.

This immunogenic mutant APC peptide appeared to be restricted to individuals harboring HLA B35:01 and HLA C03:03 (S4 Table). To test whether the peptide was responsive to either HLA type or to both, we selected an unaffected family member harboring HLA B35:01 but not HLA C03:03 (Fig 1, IV.1), and proceeded to perform a CD8^+^T cell activation assay. No significant increase in IFNγ positive CD8^+^T cells was observed in this individual (S4 Fig), suggesting that the APC mutant peptide, in all likelihood, binds and responds to cells positive for HLA C03:03. This was unexpected, given that the mutant APC peptide was predicted to bind to HLA B35:01 with four fold higher affinity than to HLA C03:03.

### Analysis of *APC* gene mutations to identify immunogenic peptides

Cancer vaccines offer significant opportunities to delay the onset of colorectal cancer in FAP affected individuals. However, immunogenic peptides derived from germline mutations in the APC gene may not be of relevance to the carrier of such a mutation due to the buildup of central tolerance, as we have observed in our study. In contrast, somatic *APC* gene mutations arising as second hits during polyp development could serve as potentially targetable sources for cancer vaccines, if proven to be immunogenic. Since the *in silico* prediction of the *APC* gene derived mutant peptide described in this study proved to be experimentally immunogenic only in normal FAP-unaffected individuals (FAP^-^, APC^WT^) but not in FAP-affected patients (FAP^-^, APC^mut^ and FAP^+^, APC^mut^), we next focused our attention on investigating the immunogenic potential of other previously documented somatic *APC* gene mutation-derived peptides, with the idea of targeting polyps harboring such somatic mutations.

We began by initially compiling a list of mutations in the APC protein from three databases: the UMD-APC (Universal mutation database-APC) database [30], the Leiden Open Variation Database (LOVD) and our proprietary database, OncoMD. These mutations may have appeared as a germline mutations in some individuals and as a second hit mutation in others. We analyzed 996 unique *APC* gene mutations using our OncoPept*VAC* neoepitope prioritization pipeline and identified 424 potential immunogenic peptides restricted to 10 HLA class-I types (Table 4). Upon experimental validation, many of these predicted immunogenic peptides could qualify as cancer vaccine candidates that could be used along with adoptive T-cell therapy to treat patients harboring specific *APC* somatic gene mutations.

**Table 4:** Immunogenic peptides from APC mutations reported from different databases. Immunogenic peptides were selected from a library of 9-mer peptides derived from non-synonymous single nucleotide variants and Indels resulting in amino acid alterations. The mutated peptides were selected on the basis of positive TCR binding and an HLA binding affinity of ≤500 nM. *OncoMD is MedGenome Labs’s proprietary database of somatic mutations. LOVD: Leiden Open (source) Variation Database UMD: Universal Mutation Database.

Databases	Genetic alterations	# number of immunogenic peptides	# variants producing immunogenic peptides	HLAs restricted to the immunogenic peptides	Unique immunogenic peptides
*OncoMD	23	4	3	3	3
LOVD	404	388	131	9	200
UMD	446	569	140	10	221

**Fig 4:**
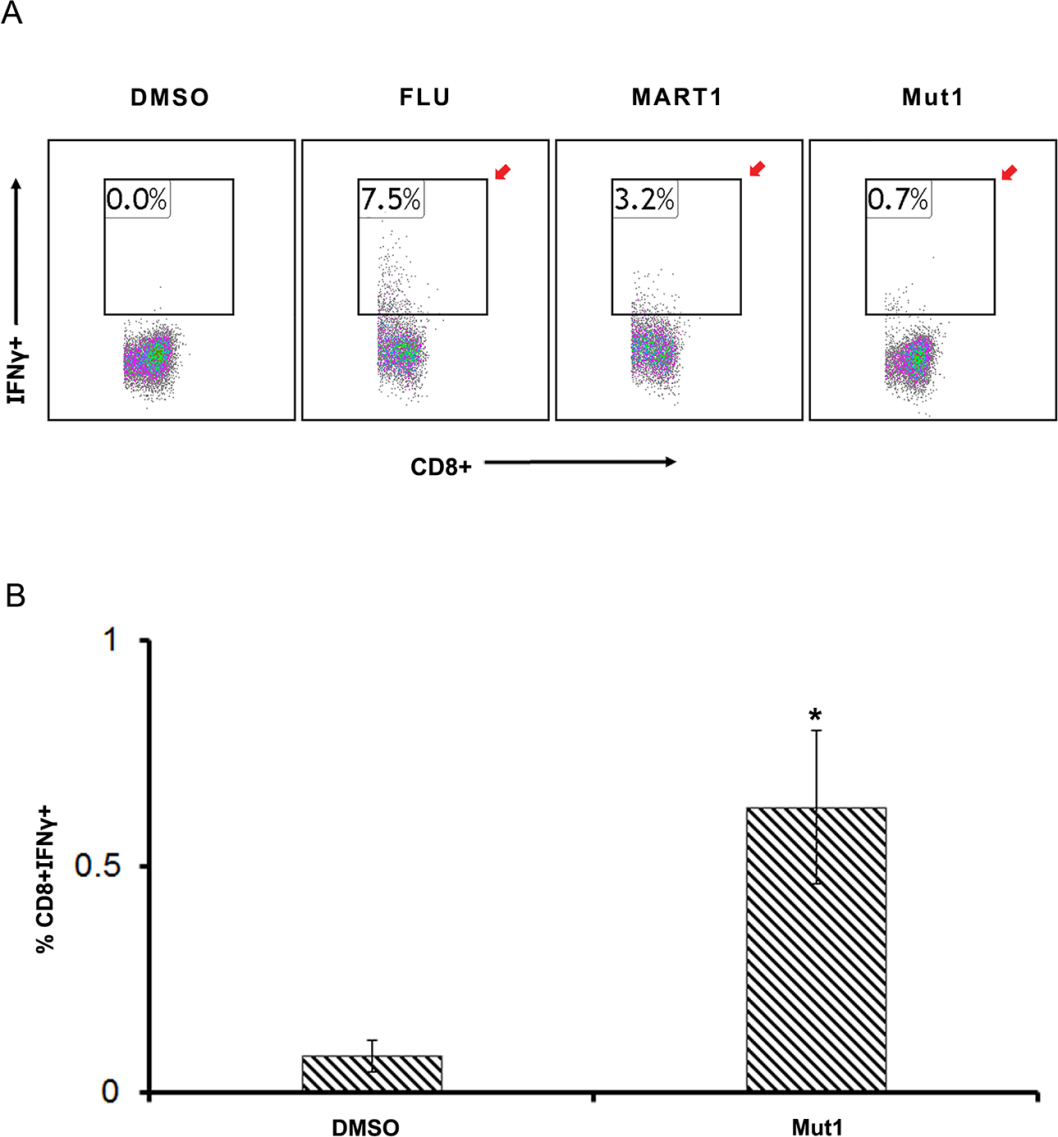
HLA A11:01 specific peptide (Mut1) derived from APC mutation p.P2018fsX26 is immunogenic. **A**. Data represents flow cytometry analysis indicating CD8^+^ IFNγ^+^ positive T cells from healthy donor 3 (HLA type in S3 Table) following exposure to Mut 1 peptide. Flu and MART1 peptides served as positive controls. Red arrows indicated an increase in CD8^+^ IFNγ^+^ positive cells **B**. Graphical representation of the Flow cytometry data in Fig 4A (*p = 0.04).

In order to test the ability of a subset of peptide candidates from our complied list of potentially inmmunogenic peptides to activate CD8+ T-cell in our *in vitro* assay, we selected six APC mutant peptides (Mut1-6) derived from mutations with high frequency of occurrence in multiple databases (Table 5) and tested them for immunogenicity using healthy donor derived immune cells. All the peptides were predicted to bind HLAs with high affinity and scored high on TCR binding as well (Table 5). However, only one of the six APC mutant peptides (Mut 1) was able to activate CD8+T cells, (Fig 4 and S5 Table: healthy donor 3). Therefore, all predicted APC mutant peptides may not be immunogenic and must be validated empirically prior to selection into a vaccine candidate pool.

**Table 5:** Details of six predicted immunogenic peptides. These peptides have been predicted to be immunogenic by Oncopept*VAC*, MedGenome Labs’s proprietary peptide prediction algorithm, and derived from the mutations that have been reported multiple times in the three databases (LOVD, UMD and OncoMD). The first number in tenth column shows the times these mutations were observed in colorectal cancer samples. The number with asterisk shows the number of times these mutations were observed in other types of cancer. These six peptides were selected for confirmation of immunogenicity in *in vitro* CD8^+^ T cell activation assays.

Gene	Variant	HLA	Wildtype Peptide	Wildtype peptide affinity (nM)		Mutant Peptide	Mutant peptide affinity (nM)	TCR binding affinity of mutant peptide	No. of times mutation observed in databases
APC	p.P2018fsX26	HLA-A11:01	LSSLSIDSE	24614.3	Mut1	SVLLVLTLK	13.71	High	2+2*
APC	p.Q1193fsX14	HLA-B35:01	IPSSQKQSF	100.41	Mut2	IPSSQKVIF	39.17	High	7+2*
APC	p.K1250fsX5	HLA-B35:01	KAATCKVSS	23571.74	Mut3	KAATCSFFY	41.35	High	4+1*
APC	p.K1250fsX5	HLA-A11:01	KAATCKVSS	20589.91	Mut3	KAATCSFFY	52.46	High	4+1*
APC	p.G1339fsX76	HLA-B08:01	TPKSPPEHY	23828.16	Mut4	HPKVHLNTM	52.46	High	5+9*
APC	p.Q541fsX19	HLA-A24:02	VIASVLRNL	13575.13	Mut5	GYCECFEEF	59.09	High	8+1*
APC	p.Q541fsX19	HLA-A24:17	VIASVLRNL	18866.1	Mut5	GYCECFEEF	69.17	High	8+1*
APC	p.V609fsX25	HLA-B35:01	SQTNTLAII	25017.04	Mut6	EPDKHFSHY	77.03	High	2+1*

## Discussion

In this study, we report a novel heterozygous mutation in the tumor suppressor *APC* gene in a family, where six members are affected with FAP and four members harbor the germline mutation, but have not developed polyps. The *APC* gene is the most frequently mutated gene in FAP [31] in which approximately 1500 loss- of- function mutations have been described. Most mutations produce truncated proteins, that have lost their ability to downregulate β-catenin levels in the cell, thereby turning on a β-catenin-mediated transcriptional program leading to increased cell proliferation and survival of colonic epithelial cells [32][33]. The *APC* gene mutation described in this study (c.735_736insT) results in a frame shifted truncated protein (p.Ser246PhefsTer6) of 250 amino acids, which retains the N-terminal homodimerization domain, but has lost all other functional domains, including the ability to bind to β-catenin. It has been reported that the location of the germline mutations in the *APC* gene determines the severity of polyposis. Highest polyp numbers are associated with mutations in the APC protein domain that binds β-catenin (codons 1250–1464), whereas mutations at the N-terminal (codons 1–157) or C-terminal (codons 1595–2843) regions of the APC protein produce fewer numbers of polyps (<100) [34]. A similar attenuated phenotype is seen with splice variants in the exon-9 region of the *APC* gene covering codons 312–412 [35]. The mutation reported in this study falls in the N-terminal region of this protein and is associated with an attenuated phenotype where <100 polyps are predicted.

The formation of benign polyps and their progression to colorectal cancer has been shown to occur in a stepwise manner, each accompanied by the accumulation of additional mutations in both tumor suppressor genes (*APC*, *p53*), oncogenes (*KRAS*) as well as in genes associated with the TGFβ pathway in individuals carrying a germline mutation in the *APC* gene. This tumor cell-intrinsic genetic alteration model predicts that a “second hit” is critical for colonic epithelial cells to undergo dysplastic growth, with an increased probability of colon cancer progression [36][37]. Despite this understanding, the use of surgery remains the mainstay of polyp treatment in FAP patients and there has been little progress in identifying therapeutics focused on blocking polyp formation and progression to CRC.

Very few studies, thus far, have examined the role of the host immune system in controlling the growth of polyps. We considered the possibility that mutations in the *APC* gene could generate immunogenic peptides, which would be recognized by the immune system as foreign, thereby controlling their growth. In this scenario, in individuals harboring the *APC* germline mutation, epithelial cells carrying the mutation would be protected from recognition by the host immune system from the very onset, due to the elimination of relevant CD8^+^ T cell clones during the establishment of central tolerance. Additional second hit somatic mutations would then be required for polyp progression. Our findings raise an important question related to the appearance of polyps in individuals in this affected family–could the appearance of a “second hit” involving a mutation in an oncogene, tumor suppressor or TGFβ pathway-related gene, work in combination with the germline *APC* gene mutation to give rise to polyps following the loss of immunosurveillance, which normally controls development and metastasis of cancers in both humans and mice? [27][28][38][39] The delayed appearance of polyps in APC^mut^ positive family members (ages 35–60) supports this notion. In the absence of polyp tissue from affected members of this FAP family, we were unable to determine the nature of the second hit mutation in either the *APC* gene itself, or in other oncogenes and tumor suppressor genes that could aid in polyp development [10]. These second hit mutations, if immunogenic could serve as potential targets for cancer vaccines.

*APC* gene mutations, which are generally early events in the development of colon cancers, can be mined for prophylactic vaccine candidates for the treatment of FAP [40]. In this regard, in a preclinical model of FAP, prophylactic vaccination targeting the *ERBB3* gene was shown to decrease polyp burden with no overt toxic effects [41].

Evidence of somatic mutations in the *APC* gene and their possible association with the progression of colorectal tumors has been proposed by Mori et al. Their study reported 43 novel somatic mutations in addition to the loss of heterozygosity mutations in the *APC* gene in 63 colorectal tumors that included FAP patients. 80% of tumors (14 adenomas and 39 carcinomas) harbored at least one mutation in the *APC* gene, of which more than 60% had incurred a second mutation [42]. In some tumors from FAP patients with a germline *APC* gene mutation, deletions were commonly observed in the inherited mutant allele but not in the wild-type APC allele. New somatic mutations have also been reported in the remaining portion of the truncated mutant allele [40]. Thus, second hits in the *APC* gene as well as other genes have been well documented in FAP.

In this study, our approach, perhaps the first of its kind, was to determine if benign polyps could be targeted by immunotherapeutic approaches to reduce polyp numbers, thereby limiting the risk of progression to CRC. To this end, we employed a combination of *in silico* neoepitope prioritization approaches followed by a CD8^+^ T cell activation assay to examine whether the mutant peptide derived from the identified frame-shifted mutant APC protein was immunogenic. Our *in silico* neoepitope prioritization pipeline (OncoPept*VAC*) identified a single 9-mer peptide to be immunogenic. The binding affinity of this mutant peptide was predicted to be 40–100 times higher for the two specific HLA Class 1 alleles, HLA-C*03:03 and HLA-B*35:01 expressed by the three FAP family members. In previous studies, differential HLA binding affinities of wildtype and mutated peptides have been shown to correlate well with CD8^+^ T cell activation in both mouse tumor models [43] as well as in many human clinical studies [27][44][45][46].

We observed a robust CD8^+^T cell response in an unaffected individual who harbored both wild-type *APC* alleles and was FAP negative (IV.9, FAP^-^/APC^wt^). On the other hand, CD8^+^T cells from both III.3 (FAP^+^/APC^mut^) and IV.2 (FAP^-^/APC^mut^) failed to respond to the mutant peptide in the CD8^+^T cell activation assay. Our observations can be summarized thus: first, that the peptide derived from the novel *APC* mutation is immunogenic and capable of selectively activating naïve CD8^+^T cells. Second, the two individuals carrying the *APC* gene mutation lacked a T cell response due to development of central tolerance.

Based on our observations, it is unlikely that immunogenic neoepitopes derived from mutated *APC* gene can be used as a vaccine in members carrying the germline *APC* mutation. However, the same mutation appearing as a second hit or somatic mutation in some FAP patients, could be targeted by this peptide. Besides vaccines, use of donor derived T cells reactive to somatic mutations may be another treatment option as demonstrated in a recent study using stage IV melanoma patients [38]. The authors of this study showed that naïve T cell repertoires from healthy blood donors provide a source of neoantigen-specific T cells, which in turn could elicit the required T-cell activation to eventually eliminate the tumor. Such an approach is however not recommended for members of our FAP family despite the observation that the unaffected family member could elicit an *APC* mutant neo-antigen specific T-cell response and CD8^+^T cells from this individual, if transferred to members harboring the germline mutation, may elicit a T-cell response leading to polyp reduction. Our recommendation is based on the fact that the germline *APC* gene mutation is in all probability expressed throughout the gastrointestinal tract, paving the way to a T-cell response even in normal tissues, which could lead to tissue destruction and have devastating consequences. Thus, cancer vaccines, or T cells reactive against specific mutations can be therapeutic options for only those FAP affected individuals who carry the mutation in their polyps (somatic mutations) and not as part of their germline DNA.

In light of this observation we surmised that identifying immunogenic peptides from somatically occurring loss-of-function mutations in the *APC* gene could be an excellent source of peptides for generating therapeutic vaccines. To this end, we compiled nearly a thousand protein altering mutations in the *APC* gene from public domain databases and our proprietary database OncoMD, and identified 424 immunogenic peptides. Many of these mutant peptides showed many orders of magnitude higher binding affinity compared to their corresponding wildtype counterparts, suggesting that they have the potential of being good vaccine candidates. Six frequently occurring predicted immunogenic peptides were chosen from this data set for empiric confirmation of their immunogenic status. However, only one of the six APC mutant peptides was capable of activating CD8+T cells harvested from a healthy donor, suggesting that all predicted APC mutant peptides are not immunogenic and must be validated experimentally prior to selection into a vaccine candidate pool. Thus, as our data show that prediction alone is insufficient to categorize peptides as vaccine candidates and that all predicted peptides candidates must be validated using immunogenicity assays.

In conclusion, we provide evidence that while a germline mutation in the *APC* gene carried by a family of FAP affected individuals is immunogenic in unaffected individuals, central tolerance mediated mechanisms likely rendered the peptide unresponsive in FAP affected members, thereby negating its use as a cancer vaccine candidate in individuals harboring this germline mutation. The occurrence of somatic *APC* gene mutations in FAP patients on the other hand, could serve as a potential source of therapeutic peptides that may delay the onset of, or prevent CRC development, through immune vigilance mechanisms over the life of an affected individual. We therefore propose that surgically removed polyps from FAP affected individuals should be tested for the presence of gene mutation-derived immunogenic peptides, which in turn must be empirically validated prior to using them as therapeutic agents to eliminate or delay the progression of FAP to colorectal cancer.

## Supporting information

S1 Fig Integrative genomics viewer (IGV) browser snapshot of the APC gene variant position (Chr5: 112136981–112136982 ins T) in three affected individuals of the FAP family (III.3 (proband), III.4, III.7). The arrow indicates the insertion of a single T nucleotide in the *APC* gene. RD: overall read depth, AF: mutant allele frequency.
(PPTX)

S2 Fig **A. The mutant APC peptide activates CD8**^**+**^ **T-cells in unrelated healthy donor 2 with HLA C03:03.** Activation of CD8^+^ T cells by the mutant APC-derived peptide from healthy donor 2 as measured by Flow cytometry of IFNγ positive cells. Healthy individual 2 (HLA in S3 Table) responded positively to the mutant APC peptide (red arrow) but not to the corresponding wildtype peptide (Table 3). **B.** Graphical representation of the Flow cytometry data of unrelated healthy donor 2 from S2A Fig. DMSO: no added peptide, Flu: positive control peptides.
(PPTX)

S3 Fig The mutant APC peptide also upregulates TNFα in FAP^-^/APC^wt^ and in a healthy unrelated donor. Top panel shows upregulation of TNFα (red arrow) in IV.9 (FAP^-^/APC^wt^) flowing exposure to the mutant APC peptide. Bottom panel represents up regulation of TNFα (red arrow) in an unrelated healthy donor. Flu peptides served as a positive control in this experiment and DMSO represents no added peptide.
(PPTX)

S4 Fig The mutant APC peptide is unable to activate CD8^+^ T-cells from donors having HLA B35:01. Data represents flow cytometry analysis IFNγ+ CD8+ T cells from IV.1 (FAP^-^/APC^wt^). HLA type of the donor: A*01:01:01:01/ A*01:01:01:01, B*35:01:01:02/ B*57:01:01, C*04:01:01:01/ C*06:02:01:01.
(PPTX)

S1 Table List of genes in the hereditary cancer panel used to screen three FAP affected individuals by NGS. (PDF)

S2 Table Summary of analysis of NGS data of the three FAP patients. (PDF)

S3 Table **A.** 9-mer peptides predicted from the identified frameshift mutation in APC protein. The five peptides were generated by altering the position of the mutant amino acid in the peptide as shown in bold. B. OncoPept*VAC* analysis revealing deprioritized mutant peptides derived from the *APC* gene mutation found in the FAP family.
(PDF)

S4 Table HLA type of three unrelated healthy donors. (PDF)

S5 Table Mutant APC derived immunogenic peptides predicted using OncoPept*VAC* and screened *in vitro* for confirmation of immunogenicity using a T cell activation assay in four healthy donors. (PDF)
