# Supplementary material for: A neoepitope derived from a novel human germline APC gene mutation in familial adenomatous polyposis shows selective immunogenicity
Source: PLoS One. 2018 Sep 26;13(9):e0203845. doi: 10.1371/journal.pone.0203845 (PMC6157866; doi:10.1371/journal.pone.0203845)
Supplement: S1 Fig — The arrow indicates the insertion of a single T nucleotide in the APC gene. RD: overall read depth, AF: mutant allele frequency. (PPTX) [file pone.0203845.s001.pptx]

## Slide 1
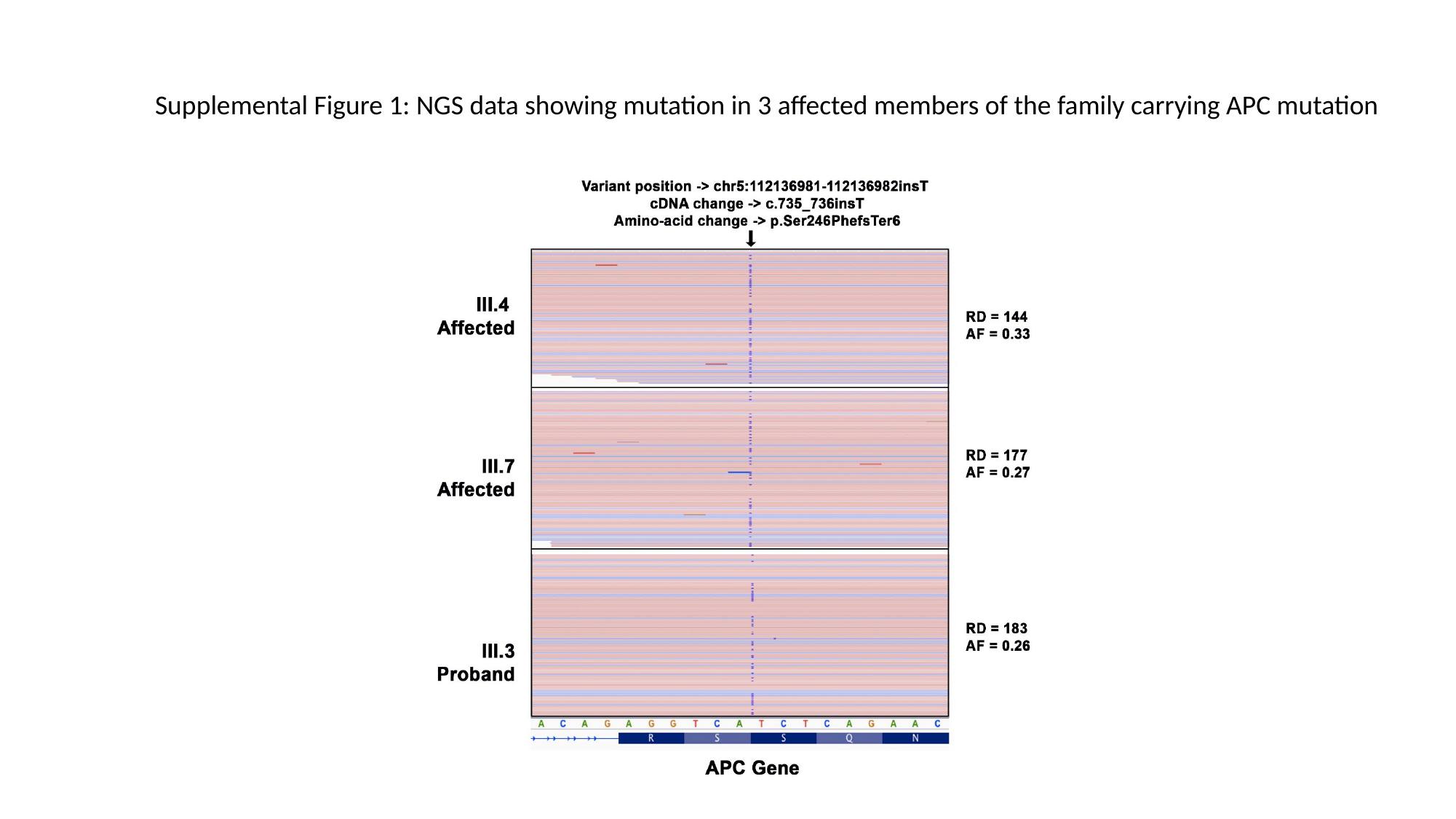

Supplemental Figure 1: NGS data showing mutation in 3 affected members of the family carrying APC mutation
