# Supplementary material for: A neoepitope derived from a novel human germline APC gene mutation in familial adenomatous polyposis shows selective immunogenicity
Source: PLoS One. 2018 Sep 26;13(9):e0203845. doi: 10.1371/journal.pone.0203845 (PMC6157866; doi:10.1371/journal.pone.0203845)
Supplement: S2 Fig — A. The mutant APC peptide activates CD8+ T-cells in unrelated healthy donor 2 with HLA C03:03. Activation of CD8+ T cells by the mutant APC-derived peptide from healthy donor 2 as measured by Flow cytometry of IFNγ positive cells. Healthy individual 2 (HLA in S3 Table) responded positively to the mutant APC peptide (red arrow) but not to the corresponding wildtype peptide (Table 3). B. Graphical representation of the Flow cytometry data of unrelated healthy donor 2 from S2A Fig. DMSO: no added peptide, Flu: positive control peptides. (PPTX) [file pone.0203845.s002.pptx]

## Slide 1
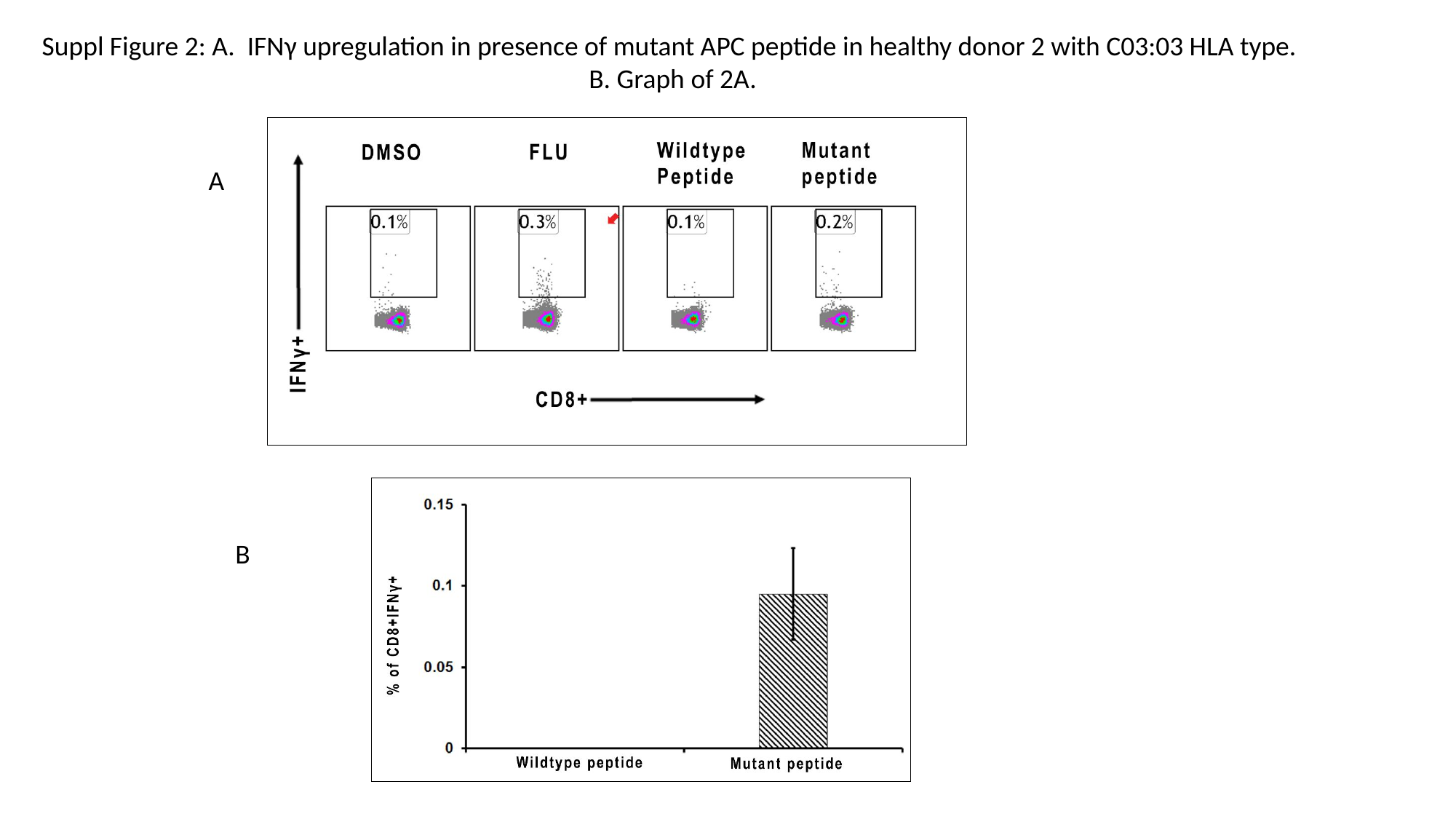

Suppl Figure 2: A. IFNγ upregulation in presence of mutant APC peptide in healthy donor 2 with C03:03 HLA type.
B. Graph of 2A.
A
B
