# Supplementary material for: A neoepitope derived from a novel human germline APC gene mutation in familial adenomatous polyposis shows selective immunogenicity
Source: PLoS One. 2018 Sep 26;13(9):e0203845. doi: 10.1371/journal.pone.0203845 (PMC6157866; doi:10.1371/journal.pone.0203845)
Supplement: S3 Fig — Top panel shows upregulation of TNFα (red arrow) in IV.9 (FAP-/APCwt) flowing exposure to the mutant APC peptide. Bottom panel represents up regulation of TNFα (red arrow) in an unrelated healthy donor. Flu peptides served as a positive control in this experiment and DMSO represents no added peptide. (PPTX) [file pone.0203845.s003.pptx]

## Slide 1
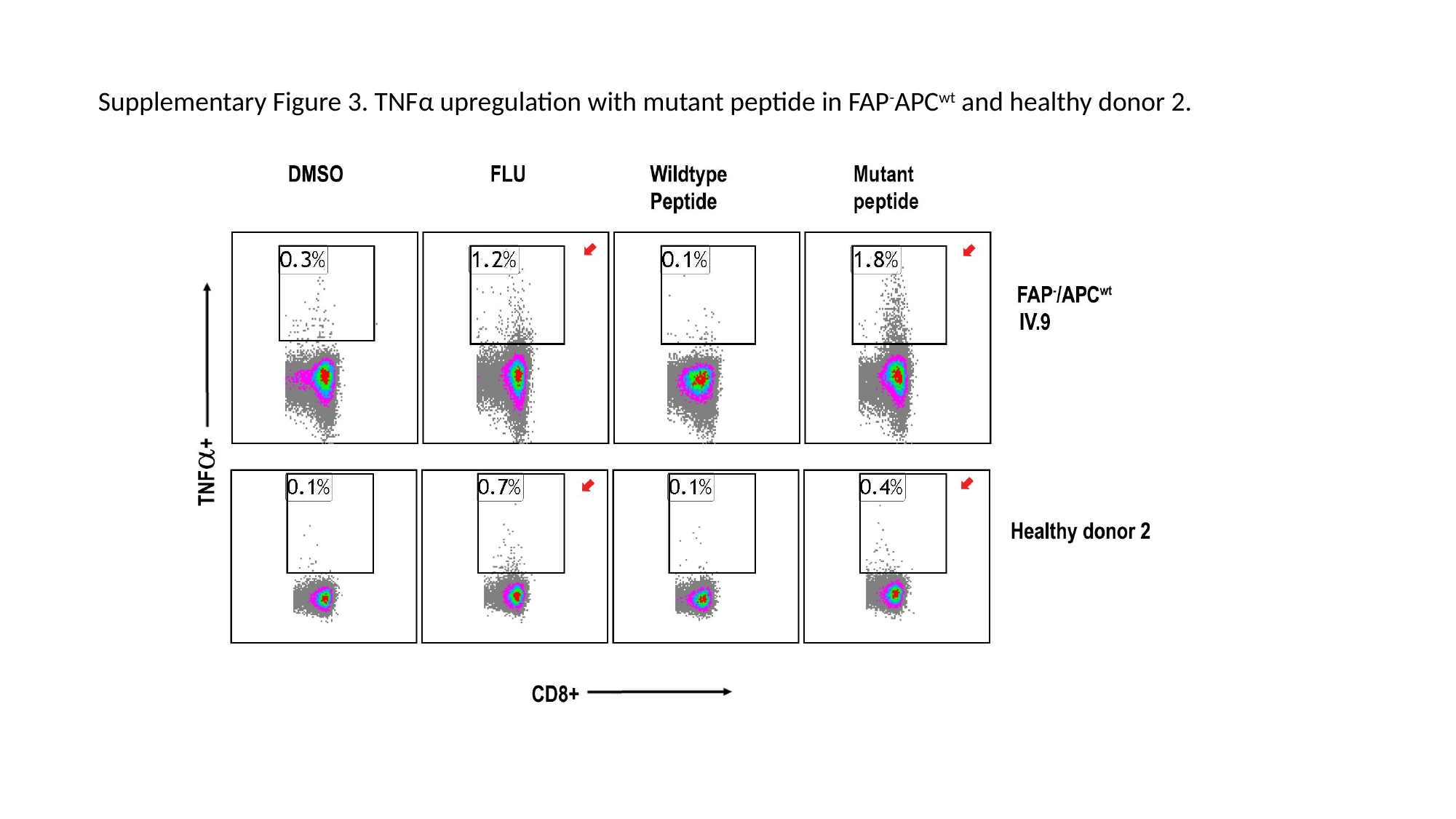

Supplementary Figure 3. TNFα upregulation with mutant peptide in FAP-APCwt and healthy donor 2.
