# Supplementary figures and images for: A neoepitope derived from a novel human germline APC gene mutation in familial adenomatous polyposis shows selective immunogenicity
Source: PLoS One. 2018 Sep 26;13(9):e0203845. doi: 10.1371/journal.pone.0203845 (PMC6157866; doi:10.1371/journal.pone.0203845)

## Slide 1
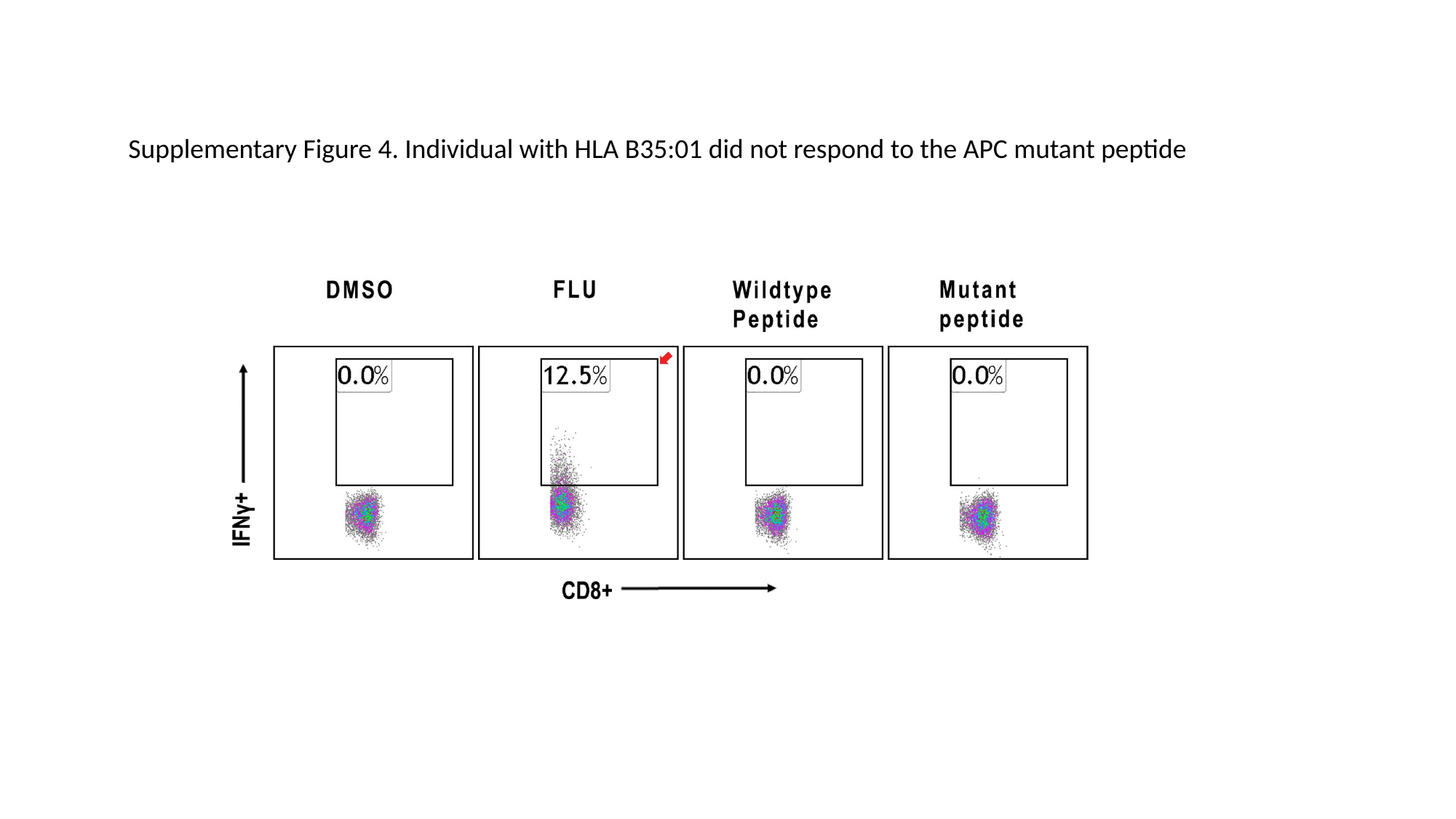

Supplementary Figure 4. Individual with HLA B35:01 did not respond to the APC mutant peptide

Supplement: S4 Fig — Data represents flow cytometry analysis IFNγ+ CD8+ T cells from IV.1 (FAP-/APCwt). HLA type of the donor: A*01:01:01:01/ A*01:01:01:01, B*35:01:01:02/ B*57:01:01, C*04:01:01:01/ C*06:02:01:01. (PPTX) [file pone.0203845.s004.pptx]
