# Supplementary material for: A neoepitope derived from a novel human germline APC gene mutation in familial adenomatous polyposis shows selective immunogenicity
Source: PLoS One. 2018 Sep 26;13(9):e0203845. doi: 10.1371/journal.pone.0203845 (PMC6157866; doi:10.1371/journal.pone.0203845)
Supplement: S1 Table — (PDF) [file pone.0203845.s005.pdf]

S1 Table. List of genes in the herediatary cancer panel used to screen three FAP affected individuals by NGS

Serial #	Gene Symbol				
1	AAAS	41	ADAMTSL2	81	ALPL
2	AARS	42	ADCK3	82	ALS2
3	AARS2	43	ADCY9	83	AMACR
4	ABAT	44	ADM	84	AMPD1
5	ABCA1	45	ADSL	85	AMT
6	ABCA4	46	AFF2	86	ANAPC5
7	ABCB7	47	AFG3L2	87	ANG
8	ABCC3	48	AGA	88	ANK2
9	ABCC6	49	AGAP2	89	ANKRD1
10	ABCC8	50	AGK	90	ANKRD11
11	ABCC9	51	AGL	91	ANO10
12	ABCD1	52	AGRN	92	ANO3
13	ABCG2	53	AGTR2	93	ANO5
14	ABCG5	54	AHI1	94	ANTXR2
15	ABCG8	55	AICDA	95	AP1S2
16	ABHD12	56	AIFM1	96	AP3B1
17	ABHD5	57	AIMP1	97	AP4B1
18	ABL1	58	AIP	98	AP4E1
19	ACAD9	59	AIPL1	99	AP4M1
20	ACADM	60	AIRE	100	AP4S1
21	ACADS	61	AK2	101	AP5Z1
22	ACADVL	62	AKAP9	102	APC
23	ACAT1	63	AKR1C3	103	APC2
24	ACAT2	64	AKT1	104	APEX1
25	ACBD5	65	AKT2	105	APOA1
26	ACO2	66	AKT3	106	APOA5
27	ACP5	67	AKT3,SDCC	107	APOB
28	ACSL4	68	ALAS2	108	APOC2
29	ACSL6	69	ALDH5A1	109	APOC3
30	ACTA1	70	ALDH7A1	110	APOE
31	ACTA2	71	ALG1	111	APTX
32	ACTB	72	ALG12	112	AR
33	ACTC1	73	ALG13	113	ARFGEF2
34	ACTG1	74	ALG2	114	ARHGEF10
35	ACTN2	75	ALG3	115	ARHGEF28
36	ACVR1	76	ALG6	116	ARHGEF6
37	ACY1	77	ALG8	117	ARHGEF9
38	ADA	78	ALG9	118	ARL13B
39	ADAM9	79	ALK	119	ARL2BP
40	ADAMTS18	80	ALMS1	120	ARL6

121	ARSA	164	BAX	207	BSCL2
122	ARSB	165	BBIP1	208	BSND
123	ARSE	166	BBS1	209	BTD
124	ARX	167	BBS10	210	BTK
125	ASAH1	168	BBS12	211	BUB1B
126	ASL	169	BBS2	212	C10orf2
127	ASPA	170	BBS4	213	C12orf65
128	ASPM	171	BBS5	214	C19orf12
129	ASS1	172	BBS7	215	C1QA
130	ASXL1	173	BBS9	216	C1QB
131	ATCAY	174	BCKDHA	217	C1QC
132	ATL1	175	BCKDHB	218	C1R
133	ATM	176	BCKDK	219	C1S
134	ATN1	177	BCL11A	220	C2
135	ATP13A2	178	BCL2	221	C21orf2
136	ATP1A3	179	BCL3	222	C2,CFB
137	ATP2A2	180	BCOR	223	C2orf71
138	ATP5E	181	BCR	224	C3
139	ATP6AP2	182	BCS1L	225	C4B
140	ATP6V1B1	183	BDNF	226	C5
141	ATP7A	184	BEAN1	227	C5orf42
142	ATP7B	185	BEST1	228	C6
143	ATP8A2	186	BICC1	229	C7
144	ATPAF2	187	BICD2	230	C8A
145	ATR	188	BIN1	231	C8B
146	ATRX	189	BIRC3	232	C8orf37
147	ATXN1	190	BIRC5	233	C9
148	ATXN10	191	BIRC6	234	C9orf72
149	ATXN2	192	BLK	235	CA2
150	ATXN3	193	BLM	236	CA4
151	ATXN7	194	BLNK	237	CA8
152	AUH	195	BLOC1S3	238	CABP4
153	AURKA	196	BLOC1S6	239	CACNA1A
154	AUTS2	197	BMP1	240	CACNA1C
155	AVPR1A	198	BMP4	241	CACNA1D
156	AXL	199	BMPR1A	242	CACNA1F
157	B4GALT1	200	BMPR2	243	CACNA1H
158	B9D1	201	BOLA3	244	CACNA2D1
159	B9D2	202	BRAF	245	CACNA2D4
160	BAG3	203	BRCA1	246	CACNB2
161	BAI3	204	BRCA2	247	CACNB4
162	BAP1	205	BRIP1	248	CALR3
163	BARD1	206	BRWD3	249	CAMTA1

250	CAPN3	293	CDC42	336	CFL2
251	CAPN5	294	CDC73	337	CFP
252	CARD11	295	CDH1	338	CFTR
253	CARD9	296	CDH23	339	CHAT
254	CARM1	297	CDH3	340	CHD7
255	CASK	298	CDHR1	341	CHD8
256	CASP10	299	CDK2	342	CHEK1
257	CASP8	300	CDK4	343	CHEK2
258	CASQ2	301	CDK5RAP2	344	CHGB
259	CATSPER2	302	CDK6	345	CHKB
260	CAV1	303	CDK7	346	CHM
261	CAV3	304	CDKL5	347	CHMP1A
262	CBFA2T3	305	CDKN1A	348	CHMP2B
263	CBL	306	CDKN1B	349	CHN1
264	CBLC	307	CDKN1C	350	CHRNA1
265	CBS	308	CDKN2A	351	CHRNA2
266	CC2D2A	309	CDKN2B	352	CHRNA4
267	CCDC50	310	CDKN2C	353	CHRNB1
268	CCDC78	311	CDKN2D	354	CHRNB2
269	CCND1	312	CDX2	355	CHRND
270	CCND2	313	CEBPA	356	CHRNE
271	CCND3	314	CEBPE	357	CHRNG
272	CCNE1	315	CEL	358	CHST14
273	CCT5	316	CENPJ	359	CIB2
274	CD19	317	CEP135	360	CIITA
275	CD247	318	CEP152	361	CISD2
276	CD27	319	CEP164	362	CIZ1
277	CD3D	320	CEP290	363	CLCN1
278	CD3E	321	CEP41	364	CLCN2
279	CD3G	322	CEP57	365	CLCN7
280	CD40	323	CEP63	366	CLDN14
281	CD40LG	324	CERK	367	CLEC4D
282	CD44	325	CERKL	368	CLEC7A
283	CD46	326	CETP	369	CLN3
284	CD55	327	CFB	370	CLN5
285	CD59	328	CFB,C2	371	CLN6
286	CD79A	329	CFD	372	CLN8
287	CD79B	330	CFH	373	CLRN1
288	CD81	331	CFHR1	374	CLTC
289	CD82	332	CFHR3	375	CNGA1
290	CD8A	333	CFHR3,CFH	376	CNGA3
291	CDAN1	334	CFHR5	377	CNGB1
292	CDC25A	335	CFI	378	CNGB3

379	CNNM4	422	COX6B1	465	D2HGDH
380	CNTN1	423	CP	466	DAG1
381	CNTNAP2	424	CPA6	467	DARS2
382	CNTNAP5	425	CPS1	468	DBH
383	COA5	426	CPT1A	469	DBT
384	COCH	427	CPT2	470	DCLRE1C
385	COG1	428	CR2	471	DCTN1
386	COG4	429	CRAT	472	DCX
387	COG5	430	CRB1	473	DDB2
388	COG6	431	CREBBP	474	DDC
389	COG7	432	CRTAP	475	DDHD1
390	COG8	433	CRX	476	DDHD2
391	COL11A1	434	CRYAB	477	DDIT3
392	COL11A2	435	CRYM	478	DDR2
393	COL18A1	436	CSF1R	479	DEPDC5
394	COL1A1	437	CSF3R	480	DES
395	COL1A2	438	CSMD3	481	DFNA5
396	COL2A1	439	CSNK1G2	482	DFNB31
397	COL3A1	440	CSRP3	483	DFNB59
398	COL4A1	441	CSTB	484	DGKB
399	COL4A3	442	CTC1	485	DGKG
400	COL4A4	443	CTDP1	486	DGKI
401	COL4A5	444	CTF1	487	DGKZ
402	COL5A1	445	CTNNA1	488	DGUOK
403	COL5A2	446	CTNNB1	489	DHCR7
404	COL6A1	447	CTNS	490	DHDDS
405	COL6A2	448	CTRC	491	DHFR
406	COL6A3	449	CTSA	492	DHTKD1
407	COL9A1	450	CTSC	493	DIABLO
408	COL9A2	451	CTSD	494	DIAPH1
409	COL9A3	452	CTSK	495	DIAPH3
410	COLEC11	453	CUL4B	496	DICER1
411	COLQ	454	CWF19L1	497	DIRAS3
412	COQ2	455	CXCR4	498	DIS3L2
413	COQ4	456	CYBA	499	DKC1
414	COQ6	457	CYBB	500	DLAT
415	COQ9	458	CYLD	501	DLD
416	CORO1A	459	CYP19A1	502	DLG3
417	COX10	460	CYP1B1	503	DLGAP2
418	COX14	461	CYP27A1	504	DLST
419	COX15	462	CYP2U1	505	DMD
420	COX20	463	CYP4V2	506	DNAJB6
421	COX4I2	464	CYP7B1	507	DNAJC19

508	DNAJC5	551	EHMT1	594	ERCC8
509	DNAJC6	552	EIF2AK3	595	ERG
510	DNM1L	553	EIF2B1	596	ERLIN2
511	DNM2	554	EIF2B2	597	ESPN
512	DNMT1	555	EIF2B3	598	ESR1
513	DNMT3B	556	EIF2B4	599	ESR2
514	DOCK4	557	EIF2B5	600	ESRRB
515	DOCK8	558	ELAC2	601	ETFA
516	DOK7	559	ELANE	602	ETFB
517	DOLK	560	ELF4	603	ETFDH
518	DOT1L	561	ELK1	604	ETHE1
519	DPAGT1	562	ELOVL2	605	ETS2
520	DPM1	563	ELOVL4	606	ETV4
521	DPM3	564	EMC1	607	EVC
522	DPP10	565	EMD	608	EVC2
523	DPP6	566	EMX2	609	EWSR1
524	DPYD	567	EN2	610	EXOC8
525	DRD1	568	ENG	611	EXOSC3
526	DRD2	569	ENO3	612	EXT1
527	DRP2	570	ENPP2	613	EXT2
528	DSC2	571	EOMES	614	EYA4
529	DSG2	572	EP300	615	EYS
530	DSP	573	EP400	616	EZH2
531	DSPP	574	EPCAM	617	F12
532	DST	575	EPG5	618	FA2H
533	DTHD1	576	EPHA2	619	FADD
534	DTNA	577	EPHA3	620	FAM126A
535	DTNB	578	EPHA8	621	FAM134B
536	DTNBP1	579	EPHB1	622	FAM161A
537	DYM	580	EPHB2	623	FANCA
538	DYNC1H1	581	EPHB4	624	FANCB
539	DYNC2H1	582	EPHB6	625	FANCC
540	DYSF	583	EPM2A	626	FANCD2
541	E2F1	584	EPO	627	FANCE
542	EARS2	585	ERBB2	628	FANCF
543	EBP	586	ERBB3	629	FANCG
544	EEF2	587	ERBB4	630	FANCI
545	EFEMP1	588	ERCC1	631	FANCL
546	EFHC1	589	ERCC2	632	FANCM
547	EFTUD2	590	ERCC3	633	FARS2
548	EGF	591	ERCC4	634	FAS
549	EGFR	592	ERCC5	635	FASLG
550	EGR2	593	ERCC6	636	FASTKD2

637	FBLN5	680	FOXO1	723	GCK
638	FBN1	681	FOXO3	724	GCLC
639	FBN2	682	FOXP1	725	GCSH
640	FBXO7	683	FOXP2	726	GDAP1
641	FBXW7	684	FOXP3	727	GDI1
642	FCGR3A	685	FOXRED1	728	GFAP
643	FCN3	686	FPR1	729	GFER
644	FERMT3	687	FRG1	730	GFI1
645	FES	688	FSCN2	731	GFM1
646	FGD1	689	FTL	732	GFM2
647	FGD4	690	FTSJ1	733	GFPT1
648	FGF14	691	FUCA1	734	GIPC3
649	FGF3	692	FURIN	735	GJA1
650	FGF8	693	FUS	736	GJA5
651	FGFR1	694	FXN	737	GJB1
652	FGFR2	695	FZD4	738	GJB2
653	FGFR3	696	G6PC	739	GJB3
654	FGFR4	697	G6PC3	740	GJB4
655	FH	698	G6PD	741	GJB6
656	FHIT	699	GAA	742	GJC1
657	FHL1	700	GAB1	743	GJC2
658	FHL2	701	GABRA1	744	GK
659	FIG4	702	GABRB3	745	GLA
660	FIGF	703	GABRD	746	GLB1
661	FKBP10	704	GABRG1	747	GLDC
662	FKBP14	705	GABRG2	748	GLI2
663	FKRP	706	GAD1	749	GLI3
664	FKTN	707	GAD2	750	GLIS3
665	FLCN	708	GALC	751	GLUD1
666	FLNA	709	GALNS	752	GM2A
667	FLNB	710	GAMT	753	GMPS
668	FLNC	711	GAN	754	GNA14
669	FLT1	712	GARS	755	GNAS
670	FLT3	713	GATA1	756	GNAT1
671	FLT4	714	GATA2	757	GNAT2
672	FLVCR1	715	GATA3	758	GNE
673	FMR1	716	GATAD1	759	GNPTAB
674	FN1	717	GATM	760	GNPTG
675	FOLR1	718	GBA	761	GNRH1
676	FOS	719	GBA2	762	GNRHR
677	FOXC1	720	GBE1	763	GNS
678	FOXG1	721	GCDH	764	GOSR2
679	FOXN1	722	GCH1	765	GPC3

766	GPD1L	809	HGF	852	IFITM5
767	GPIHBP1	810	HGSNAT	853	IFNG
768	GPR125	811	HIF1A	854	IFNGR1
769	GPR179	812	HLCS	855	IFNGR2
770	GPR56	813	HMCN1	856	IFRD1
771	GPR98	814	HMGA1	857	IFT122
772	GPSM2	815	HMGCL	858	IFT140
773	GRB2	816	HMGCS2	859	IFT43
774	GRHL2	817	HNF1A	860	IFT80
775	GRIA3	818	HNF1B	861	IGBP1
776	GRIN2A	819	HNF4A	862	IGF1R
777	GRIN2B	820	HNRNPA1	863	IGF2R
778	GRK1	821	HNRNPA2B	864	IGFBP3
779	GRM1	822	HOXA1	865	IGHMBP2
780	GRM6	823	HOXA9	866	IGLL1
781	GRN	824	HOXD10	867	IKBKAP
782	GRPR	825	HOXD11	868	IKBKG
783	GRXCR1	826	HPRT1	869	IKZF1
784	GSK3B	827	HPS1	870	IL10RA
785	GSTP1	828	HPS3	871	IL10RB
786	GSTT1	829	HPS4	872	IL12B
787	GTF2H5	830	HPS5	873	IL12RB1
788	GUCA1A	831	HPS6	874	IL17F
789	GUCA1B	832	HRAS	875	IL17RA
790	GUCY2D	833	HSD17B10	876	IL1RAPL1
791	GUSB	834	HSD17B4	877	IL1RN
792	GYG1	835	HSP90AA1	878	IL2
793	GYS1	836	HSPB1	879	IL21R
794	HADH	837	HSPB3	880	IL2RA
795	HARS	838	HSPB8	881	IL2RG
796	HARS2	839	HSPD1	882	IL36RN
797	HAX1	840	HSPG2	883	IL7R
798	HBA1	841	HUWE1	884	ILDR1
799	HBA2	842	HYAL1	885	IMMP2L
800	HCCS	843	IBA57	886	IMPDH1
801	HCN4	844	ICAM1	887	IMPG1
802	HDAC1	845	ICOS	888	IMPG2
803	HDAC2	846	IDH1	889	INPP5E
804	HDAC3	847	IDH2	890	INS
805	HDAC4	848	IDH3B	891	INSR
806	HEPACAM	849	IDS	892	INVS
807	HEXA	850	IDUA	893	IQCB1
808	HEXB	851	IER3IP1	894	IRAK4

895	IRF8	938	KCNQ3	981	LBR
896	IRS1	939	KCNQ4	982	LCA5
897	ISCU	940	KCNV2	983	LCK
898	ISPD	941	KCTD13	984	LDB3
899	ITCH	942	KCTD7	985	LDHA
900	ITGA7	943	KDM5C	986	LDLR
901	ITGB2	944	KDR	987	LDLRAD4
902	ITGB3	945	KIAA0196	988	LDLRAP1
903	ITK	946	KIAA0226	989	LEPRE1
904	ITM2B	947	KIAA1549	990	LGI1
905	ITPR1	948	KIAA2022	991	LHFPL5
906	IVD	949	KIF11	992	LHX3
907	JAG1	950	KIF1A	993	LIAS
908	JAK2	951	KIF1B	994	LIFR
909	JAK3	952	KIF5A	995	LIG1
910	JPH2	953	KIF7	996	LIG4
911	JRK	954	KIRREL3	997	LIPA
912	JUP	955	KISS1R	998	LIPC
913	KAL1	956	KIT	999	LITAF
914	KALRN	957	KLF11	1000	LMNA
915	KARS	958	KLF6	1001	LMNB1
916	KAT2B	959	KLF8	1002	LMO2
917	KAT6B	960	KLHL3	1003	LOXHD1
918	KATNAL2	961	KLHL7	1004	LPIN1
919	KBTBD13	962	KLHL9	1005	LPIN2
920	KCNA1	963	KMT2A	1006	LPL
921	KCNA5	964	KMT2C	1007	LRAT
922	KCNC3	965	KMT2D	1008	LRBA
923	KCND3	966	KRAS	1009	LRIT3
924	KCNE1	967	L1CAM	1010	LRP5
925	KCNE1L	968	L2HGDH	1011	LRPPRC
926	KCNE2	969	LAMA1	1012	LRRC8A
927	KCNE3	970	LAMA2	1013	LRRK2
928	KCNH2	971	LAMA3	1014	LRSAM1
929	KCNJ10	972	LAMA4	1015	LRTOMT
930	KCNJ11	973	LAMB2	1016	LTBP1
931	KCNJ13	974	LAMB3	1017	LYST
932	KCNJ2	975	LAMC2	1018	LZTFL1
933	KCNJ5	976	LAMC3	1019	MAGI2
934	KCNJ8	977	LAMP1	1020	MAGT1
935	KCNMA1	978	LAMP2	1021	MAK
936	KCNQ1	979	LAMTOR2	1022	MAL
937	KCNQ2	980	LARGE	1023	MAMLD1

1024	MAN2B1	1067	MGME1	1110	MT-ND1
1025	MANBA	1068	MID1	1111	MT-ND4
1026	MAOA	1069	MINPP1	1112	MT-ND5
1027	MAP2K1	1070	MKKS	1113	MT-ND5,MT-ND6
1028	MAP2K4	1071	MKS1	1114	MT-ND6
1029	MAP3K1	1072	MLC1	1115	MTO1
1030	MAPK1	1073	MLH1	1116	MTOR
1031	MAPK10	1074	MLYCD	1117	MTPAP
1032	MAPK3	1075	MMAA	1118	MTTP
1033	MAPK9	1076	MMAB	1119	MUC1
1034	MARS2	1077	MMACHC	1120	MUSK
1035	MARVELD2	1078	MMADHC	1121	MUT
1036	MASP2	1079	MMD2	1122	MUTYH
1037	MATR3	1080	MNX1	1123	MVK
1038	MAX	1081	MOGS	1124	MXI1
1039	MBD5	1082	MPDU1	1125	MYBPC1
1040	MBL2	1083	MPI	1126	MYBPC3
1041	MBTPS2	1084	MPL	1127	MYC
1042	MC2R	1085	MPO	1128	MYCL
1043	MCCC1	1086	MPV17	1129	MYCN
1044	MCCC2	1087	MPZ	1130	MYD88
1045	MCEE	1088	MR1	1131	MYF6
1046	MCM4	1089	MRE11A	1132	MYH11
1047	MCOLN1	1090	MRPS16	1133	MYH11,NDE1
1048	MCPH1	1091	MRPS22	1134	MYH14
1049	MDC1	1092	MS4A1	1135	MYH2
1050	MDM2	1093	MSH2	1136	MYH3
1051	MDM4	1094	MSH3	1137	MYH6
1052	MECOM	1095	MSH6	1138	MYH7
1053	MECP2	1096	MSMO1	1139	MYH8
1054	MED12	1097	MSR1	1140	MYH9
1055	MED25	1098	MSRB3	1141	MYL2
1056	MEF2C	1099	MST1R	1142	MYL3
1057	MEFV	1100	MSTN	1143	MYLK
1058	MEGF10	1101	MTAP	1144	MYLK2
1059	MEN1	1102	MT-ATP6	1145	MYO15A
1060	MERTK	1103	MTFMT	1146	MYO1A
1061	MET	1104	MTHFD1	1147	MYO1C
1062	MFN2	1105	MTHFR	1148	MYO1F
1063	MFRP	1106	MTHFS	1149	MYO3A
1064	MFRP,C1QTNF5	1107	MTM1	1150	MYO6
1065	MFSD8	1108	MTMR14	1151	MYO7A
1066	MGAT2	1109	MTMR2	1152	MYOT

1153	MYOZ2	1196	NEK1	1239	NR2E3
1154	MYPN	1197	NEK2	1240	NR2F1
1155	NAGA	1198	NEU1	1241	NR3C1
1156	NAGLU	1199	NEUROD1	1242	NRAS
1157	NAV3	1200	NEXN	1243	NRL
1158	NBN	1201	NF1	1244	NRXN1
1159	NCF2	1202	NF2	1245	NSD1
1160	NCF4	1203	NFKBIA	1246	NSDHL
1161	NCOA2	1204	NFU1	1247	NTNG1
1162	NCSTN	1205	NGF	1248	NTRK1
1163	NDE1	1206	NHEJ1	1249	NTRK3
1164	NDNL2	1207	NHLRC1	1250	NUBPL
1165	NDP	1208	NHP2	1251	NUP214
1166	NDRG1	1209	NHS	1252	NUP62
1167	NDUFA1	1210	NIPA1	1253	NXF5
1168	NDUFA10	1211	NIPBL	1254	NYX
1169	NDUFA11	1212	NKX2-1	1255	OAT
1170	NDUFA12	1213	NKX2-2	1256	OCLN
1171	NDUFA13	1214	NKX2-5	1257	OCRL
1172	NDUFA2	1215	NKX6-1	1258	OFD1
1173	NDUFA9	1216	NLGN3	1259	OGDH
1174	NDUFAF1	1217	NLGN4X	1260	OPA1
1175	NDUFAF2	1218	NLRP12	1261	OPA3
1176	NDUFAF3	1219	NLRP3	1262	OPHN1
1177	NDUFAF4	1220	NMNAT1	1263	OPN1LW
1178	NDUFAF5	1221	NOD2	1264	OPN1MW
1179	NDUFAF6	1222	NOP10	1265	OPN1SW
1180	NDUFAF7	1223	NOP56	1266	OPTN
1181	NDUFB3	1224	NOS1AP	1267	ORAI1
1182	NDUFB9	1225	NOS2	1268	OSTM1
1183	NDUFS1	1226	NOS3	1269	OTC
1184	NDUFS2	1227	NOTCH1	1270	OTOA
1185	NDUFS3	1228	NOTCH2	1271	OTOF
1186	NDUFS4	1229	NOTCH3	1272	OTOR
1187	NDUFS6	1230	NPC1	1273	OTUD4
1188	NDUFS7	1231	NPC2	1274	OTX2
1189	NDUFS8	1232	NPHP1	1275	OXCT1
1190	NDUFV1	1233	NPHP3	1276	PABPN1
1191	NDUFV2	1234	NPHP4	1277	PAFAH1B1
1192	NEB	1235	NPM1	1278	PAFAH1B2
1193	NEFH	1236	NPPA	1279	PAH
1194	NEFL	1237	NR1I3	1280	PAK3
1195	NEGR1	1238	NR2E1	1281	PAK7

1282	PALB2	1325	PEX11B	1368	PLCB1
1283	PANK2	1326	PEX12	1369	PLCG1
1284	PARK2	1327	PEX13	1370	PLCG2
1285	PARK7	1328	PEX14	1371	PLEC
1286	PARP1	1329	PEX16	1372	PLEKHG4
1287	PARP2	1330	PEX19	1373	PLEKHG5
1288	PAX2	1331	PEX2	1374	PLEKHM1
1289	PAX3	1332	PEX26	1375	PLG
1290	PAX4	1333	PEX3	1376	PLN
1291	PAX6	1334	PEX5	1377	PLOD1
1292	PC	1335	PEX6	1378	PLOD2
1293	PCBD1	1336	PEX7	1379	PLP1
1294	PCCA	1337	PFKM	1380	PML
1295	PCCB	1338	PFN1	1381	PMM2
1296	PCDH15	1339	PGAM2	1382	PMP22
1297	PCDH19	1340	PGK1	1383	PMS1
1298	PCDH9	1341	PGM1	1384	PMS2
1299	PCNA	1342	PGR	1385	PNKD
1300	PCNT	1343	PHF6	1386	PNKP
1301	PCSK9	1344	PHF8	1387	PNP
1302	PDE10A	1345	PHKA1	1388	PNPLA2
1303	PDE6A	1346	PHOX2B	1389	PNPLA6
1304	PDE6B	1347	PHYH	1390	PNPO
1305	PDE6C	1348	PIGA	1391	PNPT1
1306	PDE6G	1349	PIGS	1392	POLE
1307	PDE6H	1350	PIK3C3	1393	POLG
1308	PDGFA	1351	PIK3CA	1394	POLG2
1309	PDGFB	1352	PIK3CB	1395	POLR3A
1310	PDGFRA	1353	PIK3CD	1396	POLR3B
1311	PDGFRB	1354	PIK3CG	1397	POMGNT1
1312	PDHA1	1355	PIK3R1	1398	POMGNT2
1313	PDHB	1356	PIK3R2	1399	POMT1
1314	PDHX	1357	PIK3R5	1400	POMT2
1315	PDP1	1358	PINK1	1401	PON3
1316	PDSS1	1359	PIP5K1B	1402	PORCN
1317	PDSS2	1360	PITPNM3	1403	POT1
1318	PDX1	1361	PITX2	1404	POU3F4
1319	PDXK	1362	PKD1	1405	POU4F3
1320	PDYN	1363	PKD2	1406	PPARA
1321	PDZD7	1364	PKHD1	1407	PPARG
1322	PEPD	1365	PKP2	1408	PPARGC1A
1323	PEX1	1366	PLA2G5	1409	PPIB
1324	PEX10	1367	PLA2G6	1410	PPP1R13L

1411	PPP1R3A	1454	PTH1R	1497	RDH12
1412	PPP2CB	1455	PTK2	1498	RDH5
1413	PPP2R1A	1456	PTPN11	1499	RDX
1414	PPP2R1B	1457	PTPRB	1500	RECQL4
1415	PPP2R2B	1458	PTPRC	1501	REEP1
1416	PPT1	1459	PTPRD	1502	RELN
1417	PQBP1	1460	PTPRF	1503	RET
1418	PRCD	1461	PTPRQ	1504	RFT1
1419	PRF1	1462	PTRF	1505	RFX5
1420	PRICKLE1	1463	PTS	1506	RFX6
1421	PRICKLE2	1464	PUS1	1507	RFXANK
1422	PRKAG2	1465	PYGM	1508	RFXAP
1423	PRKAR1A	1466	QDPR	1509	RGR
1424	PRKCA	1467	RAB18	1510	RGS9
1425	PRKCG	1468	RAB27A	1511	RGS9BP
1426	PRKCZ	1469	RAB28	1512	RHBDF2
1427	PRKDC	1470	RAB39B	1513	RHEB
1428	PRKRA	1471	RAB3GAP1	1514	RHO
1429	PRNP	1472	RAB3GAP2	1515	RHOA
1430	PROK2	1473	RAB7A	1516	RHOH
1431	PROKR2	1474	RAC2	1517	RIMS1
1432	PROM1	1475	RAD50	1518	RLBP1
1433	PRPF3	1476	RAD51	1519	RMND1
1434	PRPF31	1477	RAD51B	1520	RNASEH2A
1435	PRPF6	1478	RAD51C	1521	RNASEH2B
1436	PRPF8	1479	RAD51D	1522	RNASEH2C
1437	PRPH	1480	RAF1	1523	RNASEL
1438	PRPH2	1481	RAG1	1524	RNASET2
1439	PRPS1	1482	RAG2	1525	RNF168
1440	PRRT2	1483	RAI1	1526	RNF170
1441	PRX	1484	RANGRF	1527	RNF216
1442	PSAP	1485	RAPSN	1528	ROGDI
1443	PSEN1	1486	RARA	1529	ROM1
1444	PSEN2	1487	RARS2	1530	ROS1
1445	PSENEN	1488	RASGRP2	1531	RP1
1446	PSMB8	1489	RAX2	1532	RP1L1
1447	PSTPIP1	1490	RB1	1533	RP2
1448	PTCH1	1491	RBCK1	1534	RP9
1449	PTCH2	1492	RBFOX1	1535	RPE65
1450	PTCHD1	1493	RBM20	1536	RPGR
1451	PTEN	1494	RBP3	1537	RPGRIP1
1452	PTF1A	1495	RBP4	1538	RPGRIP1L
1453	PTGS2	1496	RD3	1539	RPL10

1540	RPS6KA1	1583	SEMA4A	1626	SLC25A15
1541	RPS6KA2	1584	SEPN1	1627	SLC25A19
1542	RPS6KA3	1585	SEPSECS	1628	SLC25A20
1543	RPS6KB1	1586	Sep-09	1629	SLC25A22
1544	RPSA	1587	SERAC1	1630	SLC25A3
1545	RRM2B	1588	SERPINB6	1631	SLC25A38
1546	RS1	1589	SERPINF1	1632	SLC25A4
1547	RTEL1	1590	SERPING1	1633	SLC26A2
1548	RTN2	1591	SERPINH1	1634	SLC26A4
1549	RUNX1	1592	SETX	1635	SLC26A5
1550	RUNX1T1	1593	SGCA	1636	SLC2A1
1551	RYR1	1594	SGCB	1637	SLC2A10
1552	RYR2	1595	SGCD	1638	SLC2A2
1553	SACS	1596	SGCE	1639	SLC33A1
1554	SAG	1597	SGCG	1640	SLC35A1
1555	SAMHD1	1598	SGCZ	1641	SLC35C1
1556	SARS2	1599	SGSH	1642	SLC35D1
1557	SATB2	1600	SH2D1A	1643	SLC37A4
1558	SBDS	1601	SH3TC2	1644	SLC38A8
1559	SBF2	1602	SHANK2	1645	SLC39A13
1560	SCARB2	1603	SHANK3	1646	SLC39A4
1561	SCN10A	1604	SHC1	1647	SLC46A1
1562	SCN1A	1605	SHH	1648	SLC4A11
1563	SCN1B	1606	SHROOM4	1649	SLC6A1
1564	SCN2A	1607	SIAE	1650	SLC6A19
1565	SCN3B	1608	SIGMAR1	1651	SLC6A3
1566	SCN4A	1609	SIL1	1652	SLC6A4
1567	SCN4B	1610	SIX1	1653	SLC6A8
1568	SCN5A	1611	SIX3	1654	SLC8A1
1569	SCN8A	1612	SKI	1655	SLC9A6
1570	SCN9A	1613	SKIV2L	1656	SLC9A9
1571	SCO1	1614	SKP2	1657	SLCO1B1
1572	SCO2	1615	SLC12A1	1658	SLX4
1573	SCP2	1616	SLC12A6	1659	SMAD2
1574	SDCCAG8	1617	SLC16A2	1660	SMAD3
1575	SDHA	1618	SLC17A5	1661	SMAD4
1576	SDHAF1	1619	SLC17A8	1662	SMARCA4
1577	SDHAF2	1620	SLC19A2	1663	SMARCAL1
1578	SDHB	1621	SLC19A3	1664	SMARCB1
1579	SDHC	1622	SLC1A3	1665	SMC1A
1580	SDHD	1623	SLC22A5	1666	SMG6
1581	SEC23B	1624	SLC24A1	1667	SMN1
1582	SEMA3A	1625	SLC25A12	1668	SMN2

1669	SMN2,SMN1	1712	SREBF2	1755	TBL1X
1670	SMO	1713	SRGAP2	1756	TBP
1671	SMPD1	1714	SRL	1757	TBX1
1672	SMPX	1715	SRPX2	1758	TBX20
1673	SMS	1716	SSPN	1759	TBXAS1
1674	SNAP29	1717	ST3GAL3	1760	TCAP
1675	SNCA	1718	ST7	1761	TCF12
1676	SNCG	1719	STAT1	1762	TCF21
1677	SNRNP200	1720	STAT2	1763	TCF3
1678	SNRPN	1721	STAT3	1764	TCF4
1679	SNTA1	1722	STAT4	1765	TCIRG1
1680	SNTB1	1723	STAT5B	1766	TCN2
1681	SNTB2	1724	STIL	1767	TCTN1
1682	SNX10	1725	STIM1	1768	TCTN2
1683	SOCS1	1726	STK11	1769	TCTN3
1684	SOCS2	1727	STK3	1770	TDP1
1685	SOD1	1728	STK4	1771	TEAD1
1686	SOS1	1729	STRC	1772	TECTA
1687	SOX10	1730	STUB1	1773	TEK
1688	SOX2	1731	STX11	1774	TERC
1689	SOX3	1732	STXBP1	1775	TERT
1690	SOX5	1733	STXBP2	1776	TET2
1691	SOX9	1734	SUCLA2	1777	TFE3
1692	SP1	1735	SUCLG1	1778	TFG
1693	SP110	1736	SUFU	1779	TGFB1
1694	SP7	1737	SUMF1	1780	TGFB2
1695	SPAST	1738	SUOX	1781	TGFB3
1696	SPATA7	1739	SURF1	1782	TGFBR1
1697	SPEN	1740	SYN1	1783	TGFBR2
1698	SPG11	1741	SYNE1	1784	TGIF1
1699	SPG20	1742	SYP	1785	TGM6
1700	SPG21	1743	SYT14	1786	TH
1701	SPG7	1744	TACO1	1787	THAP1
1702	SPINK1	1745	TACR3	1788	THBD
1703	SPINK5	1746	TAF1	1789	THBS1
1704	SPP1	1747	TAL1	1790	TICAM1
1705	SPR	1748	TAP1	1791	TIMM44
1706	SPRY2	1749	TAP2	1792	TIMM8A
1707	SPTAN1	1750	TAPBP	1793	TIMP3
1708	SPTBN2	1751	TARDBP	1794	TINF2
1709	SPTLC1	1752	TAZ	1795	TJP2
1710	SPTLC2	1753	TBC1D24	1796	TK1
1711	SRC	1754	TBCE	1797	TK2

1798	TLR3	1841	TPP1	1884	TYROBP
1799	TLX1	1842	TPR	1885	UBA1
1800	TMC1	1843	TPRN	1886	UBE2A
1801	TMC6	1844	TRAF3	1887	UBE3A
1802	TMC8	1845	TRDN	1888	UBQLN2
1803	TMEM126A	1846	TREM2	1889	UNC119
1804	TMEM127	1847	TREX1	1890	UNC13D
1805	TMEM138	1848	TRIM32	1891	UNC93B1
1806	TMEM216	1849	TRIOBP	1892	UNG
1807	TMEM231	1850	TRIP11	1893	UPF3B
1808	TMEM237	1851	TRMU	1894	UQCRB
1809	TMEM38B	1852	TRPM1	1895	UQCRC2
1810	TMEM43	1853	TRPM4	1896	UQCRQ
1811	TMEM67	1854	TRPV4	1897	UROC1
1812	TMEM70	1855	TRRAP	1898	USB1
1813	TMIE	1856	TSC1	1899	USH1C
1814	TMPO	1857	TSC2	1900	USH1G
1815	TMPRSS3	1858	TSEN2	1901	USH2A
1816	TMPRSS5	1859	TSEN34	1902	VAMP1
1817	TNFRSF11A	1860	TSEN54	1903	VAPB
1818	TNFRSF13B	1861	TSFM	1904	VCAN
1819	TNFRSF13C	1862	TSPAN12	1905	VCL
1820	TNFRSF1A	1863	TSPAN7	1906	VCP
1821	TNFSF11	1864	TTBK2	1907	VDAC1
1822	TNK2	1865	TTC19	1908	VDR
1823	TNNC1	1866	TTC21B	1909	VEGFA
1824	TNNI2	1867	TTC37	1910	VHL
1825	TNNI3	1868	TTC8	1911	VLDLR
1826	TNNT1	1869	TTN	1912	VPS13A
1827	TNNT2	1870	TTPA	1913	VPS13B
1828	TNNT3	1871	TTR	1914	VPS35
1829	TNPO3	1872	TUBA1A	1915	VPS37A
1830	TNXB	1873	TUBA4A	1916	VPS54
1831	TOP2A	1874	TUBA8	1917	VRK1
1832	TOPORS	1875	TUBA8,PEX	1918	WAS
1833	TOR1A	1876	TUBB2B	1919	WDPCP
1834	TP53	1877	TUBB3	1920	WDR19
1835	TP73	1878	TUBB4A	1921	WDR35
1836	TPK1	1879	TUBGCP6	1922	WDR62
1837	TPM1	1880	TUFM	1923	WDR81
1838	TPM2	1881	TULP1	1924	WFS1
1839	TPM3	1882	TYK2	1925	WIPF1
1840	TPO	1883	TYMP	1926	WNK1

1927	WNT1
1928	WRAP53
1929	WRN
1930	WT1
1931	XIAP
1932	XK
1933	XPA
1934	XPC
1935	XPNPEP3
1936	YARS
1937	YARS2
1938	YWHAE
1939	ZAP70
1940	ZBTB24
1941	ZCCHC12
1942	ZDHHC15
1943	ZDHHC9
1944	ZEB2
1945	ZFP57
1946	ZFYVE26
1947	ZFYVE27
1948	ZIC1
1949	ZIC2
1950	ZIC4
1951	ZNF331
1952	ZNF41
1953	ZNF423
1954	ZNF507
1955	ZNF513
1956	ZNF592
1957	ZNF674
1958	ZNF711
1959	ZNF804A
1960	ZNF81
1961	ZNHIT6
