# Supplementary material for: A neoepitope derived from a novel human germline APC gene mutation in familial adenomatous polyposis shows selective immunogenicity
Source: PLoS One. 2018 Sep 26;13(9):e0203845. doi: 10.1371/journal.pone.0203845 (PMC6157866; doi:10.1371/journal.pone.0203845)
Supplement: S2 Table — (PDF) [file pone.0203845.s006.pdf]

**S2 Table . Summary of analysis of NGS data of the three FAP patients**			
Sample ID/Name	III.7	III.3	III.4
# of reads	202,27,514	177,20,788	163,12,114
Total data (Gb)	2.02	1.77	1.63
Read length (bp)	100	100	100
Data >= Q30 (%)	93.7	93.89	93.45
Alignment (%)	99.44	99.13	99.18
Median mapping quality (Phred)	60	60	60
Target region coverage (%)	99.3	99.3	99.31
Read duplicate (%)	4.37	3.42	5.93
Read on-target (%)	82.84	83.05	83.04
Avg. read depth (X)	175.13	155.99	138.05
