# Supplementary material for: A neoepitope derived from a novel human germline APC gene mutation in familial adenomatous polyposis shows selective immunogenicity
Source: PLoS One. 2018 Sep 26;13(9):e0203845. doi: 10.1371/journal.pone.0203845 (PMC6157866; doi:10.1371/journal.pone.0203845)
Supplement: S3 Table — A. 9-mer peptides predicted from the identified frameshift mutation in APC protein. The five peptides were generated by altering the position of the mutant amino acid in the peptide as shown in bold. B. OncoPeptVAC analysis revealing deprioritized mutant peptides derived from the APC gene mutation found in the FAP family. (PDF) [file pone.0203845.s007.pdf]

**S3 Table A. 9-mer peptides predicted from the frameshift mutation in APC protein. The five peptides were generated by altering the position of the mutant amino acid in the peptide as shown in bold.**		
Sequence number	Wildtype peptide sequence	Mutant peptide sequence
Seq-1	QATEAERS**S**	QATEAERS**F**
Seq-2	ATEAERS**SQ**	ATEAERS**FS**
Seq-3	TEAERS**SQN**	TEAERS**FSE**
Seq-4	EAERS**SQNK**	EAERS**FSEQ**
Seq-5	AERS**SQNKH**	AERS**FSEQA**

S3 Table B. OncoPeptVAC analysis revealing deprioritized mutant peptides derived from the APC gene mutation found in the FAP family.

HLA type	Seq ID	Wildtype peptide seq	Wildtype peptide Affinity (nM)	Mutant Peptide seq	Mutant peptide Affinity (nM)
B35:01	Seq2	ATEAERSSQ	39408.75	ATEAERSFS	40489.28
C03:03		ATEAERSSQ	39196.13	ATEAERSFS	39837.46
B35:01	Seq3	TEAERSSQN	37535.94	TEAERSFSE	29425.27
C03:03		TEAERSSQN	39196.13	TEAERSFSE	38150.11
B35:01	Seq4	EAERSSQNK	34237.82	EAERSFSEQ	19717.81
C03:03		EAERSSQNK	37333.42	EAERSFSEQ	32085.75
B35:01	Seq5	AERSSQNKH	37132	AERSFSEQA	38150.11
C03:03		AERSSQNKH	40929.75	AERSFSEQA	39196.13
