# Supplementary material for: A neoepitope derived from a novel human germline APC gene mutation in familial adenomatous polyposis shows selective immunogenicity
Source: PLoS One. 2018 Sep 26;13(9):e0203845. doi: 10.1371/journal.pone.0203845 (PMC6157866; doi:10.1371/journal.pone.0203845)
Supplement: S4 Table — (PDF) [file pone.0203845.s008.pdf]

S4 Table. HLA type of three unrelated healthy donors.	
Donor	HLA type
Healthy donor 1	A*02:01:01G/A*68:12, B*15:01:01G/B*44:05:01, C*02:02:02G/C*03:03:01G
Healthy donor 2	A*24:02:01:01/A*24:02:01:01, B*15:05:01/B*51:01:01:01, C*03:03:01/C*16:02:01
Healthy donor 3	A*02:11:01/A*11:01:01:01, B*35:01:01:02/B*35:03:01:01, C*04:01:01:01/C*12:03:01:01
