# Supplementary material for: A neoepitope derived from a novel human germline APC gene mutation in familial adenomatous polyposis shows selective immunogenicity
Source: PLoS One. 2018 Sep 26;13(9):e0203845. doi: 10.1371/journal.pone.0203845 (PMC6157866; doi:10.1371/journal.pone.0203845)
Supplement: S5 Table — (PDF) [file pone.0203845.s009.pdf]

S5 Table. Mutant APC derived immunogenic peptides predicted using OncoPeptVAC and screened in vitro for confirmation of immunogenicity using a T cell activation assay in four healthy donors.

Healthy donor 3: HLA A11:01, B35:01

APC peptide screening — HLA A11:01

		%CD8+IFNγ+				%CD8+TNFα+			
		Replicate 1	Replicate 2	Replicate 3	Avg	Replicate 1	Replicate 2	Replicate 3	Avg
	DMSO	0.04	0.09	0.11	0.08	0.11	0.16	0.26	0.18
	flu	9.49	6.31	7.65	7.82	2.01	0.99	2.33	1.78
	MelA	2.08	2.6	3.32	2.67	0.5	0.33	0.48	0.44
SVLLVLTLK	Mut 1	0.75	0.71	0.44	0.63	1.05	1.11	1.07	1.08
IPSSQKVIF	Mut 2	0.11	0.09	0.17	0.12	0.19	0.23	0.22	0.21
KAATCSFFY	Mut 3	0.04	0.12	0.07	0.08	0.1	0.12	0.07	0.10
EPDKHFSHY	Mut 6	0.05	0.04	0.04	0.04	0.14	0.2	0.04	0.13
IPSSQKQSF	WT 2	0.03	0.18	0.08	0.10	0.1	0.21	0.1	0.14
KAATCKVSS	WT 3	0.04	0.1	0.04	0.06	0.11	0.19	0.13	0.14

Healthy donor 4: HLA A11:01, B35:01

		%CD8+IFNγ+				%CD8+TNFα+			
		Replicate 1	Replicate 2	Replicate 3	Avg	Replicate 1	Replicate 2	Replicate 3	Avg
	DMSO	0.3	0.2	0.6	0.37	0.6	0.9	1.3	0.93
	flu	13.5	8.3	12.3	11.37	11	11.1	10.3	10.8
SVLLVLTLK	Mut 1	0.7	0.6	0.3	0.53	0.9	0.9	0.7	0.83
IPSSQKVIF	Mut 2	0.2	0	0.3	0.17	0.3	0.1	0.4	0.27
KAATCSFFY	Mut 3	0.2	0.1	0.2	0.17	0.3	0.3	0.5	0.37
EPDKHFSHY	Mut 6	0.2	0.1	0.3	0.20	0.2	0.7	0.9	0.60
IPSSQKQSF	WT 2	0.3	0.1	0.2	0.20	0.5	0.5	0.4	0.47
KAATCKVSS	WT 3	0.2	0.2	0.5	0.30	0.3	0.1	0.6	0.33

Healthy donor 5: A24:02, B08:01

		%CD8+IFNγ+				%CD8+TNFα+			
		Replicate 1	Replicate 2	Replicate 3	Avg	Replicate 1	Replicate 2	Replicate 3	Avg
	DMSO	0.2	0	0.1	0.10	1.2	0.5	0.5	0.73
	flu	8.8	7.5	6.4	7.57	6.1	5.5	5.3	5.63
HPKVHLNTM	Mut 4	0.1	0.2	0.1	0.13	0.8	0.5	0.2	0.50
GYCECFEEF	Mut 5	0.1	0.1	0.1	0.10	1.1	0.6	0.4	0.70

Healthy donor 6: A24:02, B08:01

		%CD8+IFNγ+				%CD8+TNFα+			
		Replicate 1	Replicate 2	Replicate 3	Avg	Replicate 1	Replicate 2	Replicate 3	Avg
	DMSO	0.1	0	0	0.03	0.5	0.9	0.4	0.60
	flu	14.8	14.9	12.5	14.07	3.2	5.8	2	3.67
HPKVHLNTM	Mut 4	0.1	0.1	0.1	0.10	0.9	0.8	1.1	0.93
GYCECFEEF	Mut 5	0.2	0.3	0.1	0.20	0.5	0.5	0.4	0.47
